# TREX1 Deficiency Induces ER Stress-Mediated Neuronal Cell Death by Disrupting Ca^2+^ Homeostasis

**DOI:** 10.1007/s12035-021-02631-3

**Published:** 2022-01-07

**Authors:** Debasish Halder, Su-Jin Jeon, Ji-Yong Yoon, Jeong-Ju Lee, Soo Young Jun, Min-Hyuk Choi, Bohyeon Jeong, Duk Hyun Sung, Da Yong Lee, Byoung Joon Kim, Nam-Soon Kim

**Affiliations:** 1 Rare Disease Research Center, Korea Research Institute of Bioscience and Biotechnology (KRIBB), 34141 Daejeon, Republic of Korea; 2 Genome Research Center, Korea Research Institute of Bioscience and Biotechnology, 34141 Daejeon, Republic of Korea; 3 Department of Functional Genomics, KRIBB School of Bioscience, University of Science and Technology, 34113 Daejeon, Republic of Korea; 4 Department of Physical and Rehabilitation Medicine, Sungkyunkwan University School of Medicine, Samsung Medical Center, 06351 Seoul, Republic of Korea; 5 Department of Neurology, Sungkyunkwan University School of Medicine, Samsung Medical Center, 06351 Seoul, Republic of Korea

**Keywords:** Three prime repair exonuclease 1, ER stress, BiP/GRP78, Ca^2+^ homeostasis, Neuronal cells, Hereditary spastic paraplegia

## Abstract

TREX1 is an exonuclease that degrades extranuclear DNA species in mammalian cells. Herein, we show a novel mechanism by which TREX1 interacts with the BiP/GRP78 and TREX1 deficiency triggers ER stress through the accumulation of single-stranded DNA and activates unfolded protein response (UPR) signaling via the disruption of the TREX1-BiP/GRP78 interaction. In TREX1 knockdown cells, the activation of ER stress signaling disrupted ER Ca^2+^ homeostasis via the ERO1α-IP3R1-CaMKII pathway, leading to neuronal cell death. Moreover, TREX1 knockdown dysregulated the Golgi-microtubule network through Golgi fragmentation and decreased Ac-α-tubulin levels, contributing to neuronal injury. These alterations were also observed in neuronal cells harboring a TREX1 mutation (V91M) that has been identified in hereditary spastic paraplegia (HSP) patients in Korea. Notably, this mutation leads to defects in the TREX1-BiP/GRP78 interaction and mislocalization of TREX1 from the ER and possible disruption of the Golgi-microtubule network. In summary, the current study reveals TREX1 as a novel regulator of the BiP/GRP78 interaction and shows that TREX1 deficiency promotes ER stress-mediated neuronal cell death, which indicates that TREX1 may hold promise as a therapeutic target for neurodegenerative diseases such as HSP.

**Supplementary Information:**

The online version contains supplementary material available at 10.1007/s12035-021-02631-3.

## Introduction

Three prime repair exonuclease 1 (TREX1) is a major 3′ DNA exonuclease that degrades single- and double-stranded DNA polymers in mammalian cells. The N-terminal catalytic domain (242 amino acids) of TREX1 acts on extranuclear DNA species to prevent self-DNA from activating the interferon response. The C-terminal 72 amino acids contain a hydrophobic region that localizes TREX1 to the endoplasmic reticulum (ER) in the perinuclear space of cells and plays an essential role in protein folding/biosynthesis [1–4]. The major role of TREX1 is degradation of single-stranded DNA (ssDNA) that is derived from endogenous retroelements and HIV DNA that is generated during HIV-1 infection, thereby preventing activation of the cell-intrinsic autoimmune pathway. Mutations in human TREX1 have been linked to a broad spectrum of autoimmune diseases, including Aicardi-Goutieres syndrome, familial chilblain lupus, systemic lupus erythaematosus, and retinal vasculopathy [5, 6]. Growing evidence also suggests that TREX1 deficiency causes other diseases, such as cellular senescence, cardiomyopathy, and cancer, through the regulation of different mechanisms [7–11].

TREX1 localizes to the ER, which is an essential cellular organelle critical for calcium (Ca^2+^) homeostasis. In eukaryotes, disruption of ER homeostasis induces ER stress, which results in the dissociation of the ER chaperone BiP (also known as GRP78) from ER stress sensors, leading to unfolded protein response (UPR) activation [12]. During prolonged ER stress, Ca^2+^ release from the ER quickly affects ER Ca^2+^ homeostasis and various cellular functions that have been found to be impaired in many pathophysiological processes, including Alzheimer’s disease, Parkinson’s disease, Huntington’s disease, and amyotrophic lateral sclerosis [13–15]. Recent evidence has suggested a major role of TREX1 in the Ca^2+^ signaling process [10]. In particular, the inhibition of TREX1 by miRNA-103 dysregulates the L-type Ca^2+^ channel CACNA1C, leading to the induction of IFN responses [11]. Considering these findings, it seems likely that TREX1 deficiency has an altering effect on ER Ca^2+^ homeostasis and is thus associated with neurodegenerative disorders; however, these connections have been largely unexplored.

Hereditary spastic paraplegia (HSP) is a large group of genetic neurologic disorders that primarily affect the axons of corticospinal motor neurons and lead to the symptoms of progressive lower limb spasticity and weakness. Among the pathogenic mechanisms underlying axonopathy in HSP, impaired ER function and altered ER-microtubule contact have been widely studied [16–19]. In neuronal cells, the ER forms a stable network of microtubules that plays an important role in microtubule stabilization, which is vital for the extension of axon length. However, modulation of ER function destabilizes microtubule dynamics by inducing either severe defects in α-tubulin acetylation or the loss of α-tubulin acetylation, a marker of stable microtubules, thereby causing axonopathy [20]. Notably, mutations in HSP genes, which are commonly observed in many neurodegenerative diseases, have been implicated in defects in Golgi morphology, a potent microtubule-organizing organelle that plays major roles in microtubule dynamics and organization [21, 22].

In this study, we reveal a new mechanistic role for TREX1 mediated through the ER stress-induced release of Ca^2+^ and subsequent induction of apoptosis; this mechanism of TREX1 has not been previously reported. We show that TREX1 is an important ER stress regulator that triggers ER stress through the accumulation of ssDNA in the ER and activates UPR signaling via the disruption of the TREX1-BiP/GRP78 interaction. Moreover, we demonstrate that a genetic mutation in the TREX1 gene, which has been identified in a Korean family with HSP, leads to neurodegeneration via alteration of the Ca^2+^ homeostasis pathway in the ER, and disruptions in the Golgi-microtubule network have also been observed. Thus, the present study reveals a new mechanism by which TREX1 underlies the pathology of TREX1-related diseases.

## Results

### TREX1 Knockdown Inhibits Neuronal Differentiation and Triggers Neuronal Cell Death

**Fig. 1:**
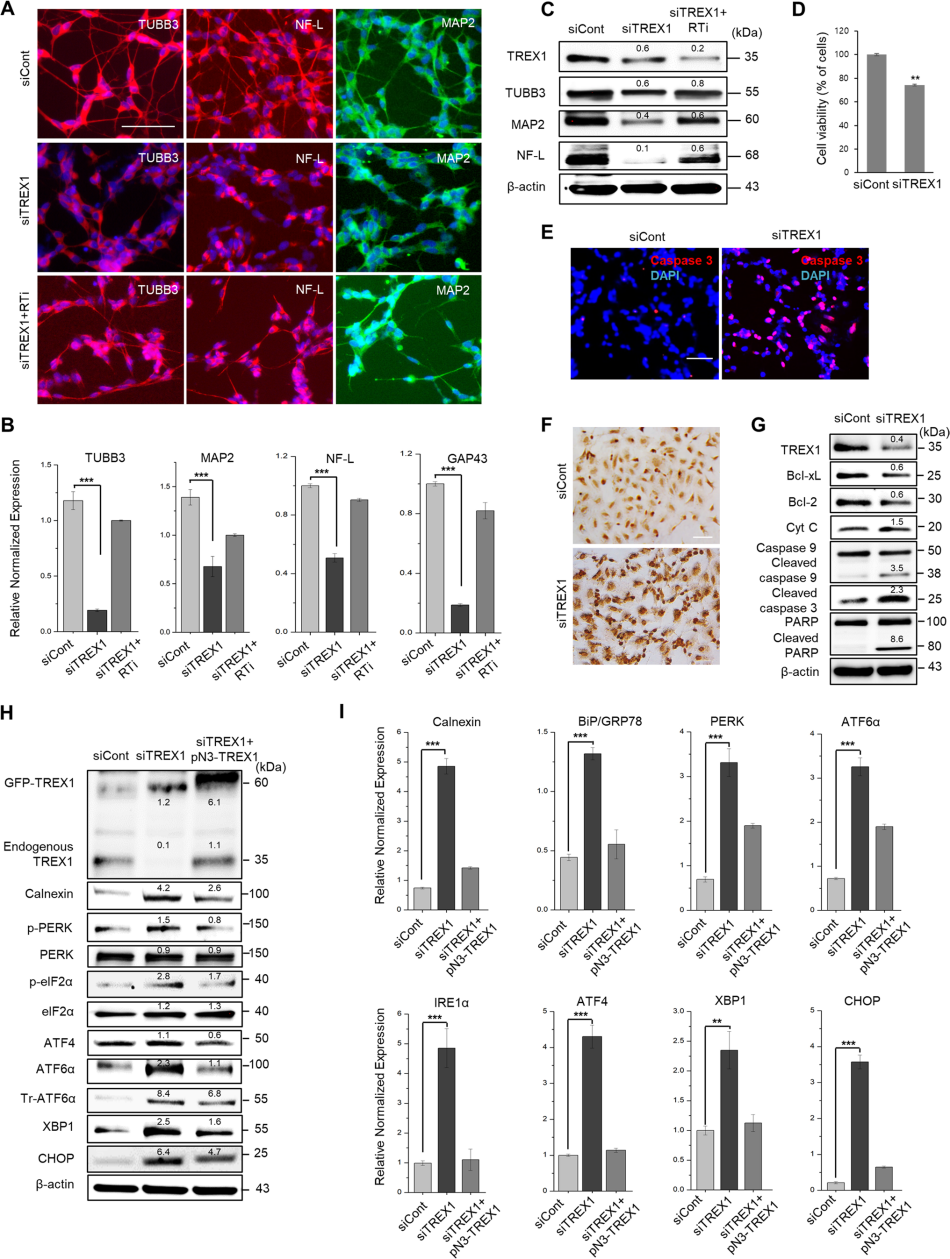
TREX1 deficiency induces ER stress and the UPR and triggers neuronal cell death. Human SH-SY5Y neuroblastoma cells were transfected with TREX1-specific siRNA (siTREX1) and control siRNA (siCont). The transfected cells were allowed to differentiate for 2–3 days in the presence of RA. Where indicated, the effects of TREX1 silencing were reversed by treating siTREX1 cells with either the wild-type TREX1 plasmid (pN3-TREX1) or reverse transcriptase inhibitors such as 3TC and d4T. **A** The differentiated cells were immunostained with neuron-specific markers, such as TUBB3, MAP2, and NF-L. Scale bar: 50 µm. **B**, **C** The mRNA and protein levels of the neuron-specific markers were examined by using quantitative RT-PCR and western blot analyses, respectively. **D** The cell viability of TREX1 knockdown cells. **E**, **F** The apoptosis process was examined by immunostaining with an anti-cleaved caspase 3 antibody (**E**) and TUNEL assay (**F**). **G** The expression of apoptotic pathway proteins was determined by western blot analyses. **H** Western blot analyses of UPR signaling proteins. **I** The mRNA levels of UPR pathway genes were analyzed by quantitative RT-PCR. The error bars show the SEM. **, *p* < 0.01; ***, *p* < 0.001; we employed one-way ANOVA test for significant analysis of data in multigroup. The data shown in all the panels are representative of three independent experiments

To determine whether the loss of TREX1 affects neuronal development, we knocked down the TREX1 gene in SH-SY5Y neuroblastoma cells and examined neuronal differentiation by immunocytochemical analysis of neuron-specific markers, such as TUBB3, MAP2, and NF-L. The results showed that TREX1 knockdown inhibited the expression of neuronal markers compared to that of control cells (Fig. 1A–C). However, TREX1-silenced neuronal cells treated with nucleoside analog reverse transcriptase inhibitors (RTis) [5, 23] lamivudine (3TC) and stavudine (d4T) were relatively healthier with longer neurites than TREX1-silenced cells that were not treated with a RTi (Fig. 1A; bottom panel and Fig. 1B and 1C; right panel). TREX1 knockdown resulted in a decrease of cell viability (Fig. 1D). In addition, TREX1-silenced neuronal cells exhibited an increase in caspase 3- and TUNEL-positive cells compared with control cells (Fig. 1E and 1F). Moreover, TREX1 silencing induced the expression of apoptotic pathway proteins (Fig. 1G). Taken together, these results indicate that the silencing of TREX1 impairs neuronal development and primes cells for apoptosis.

**Fig. 2:**
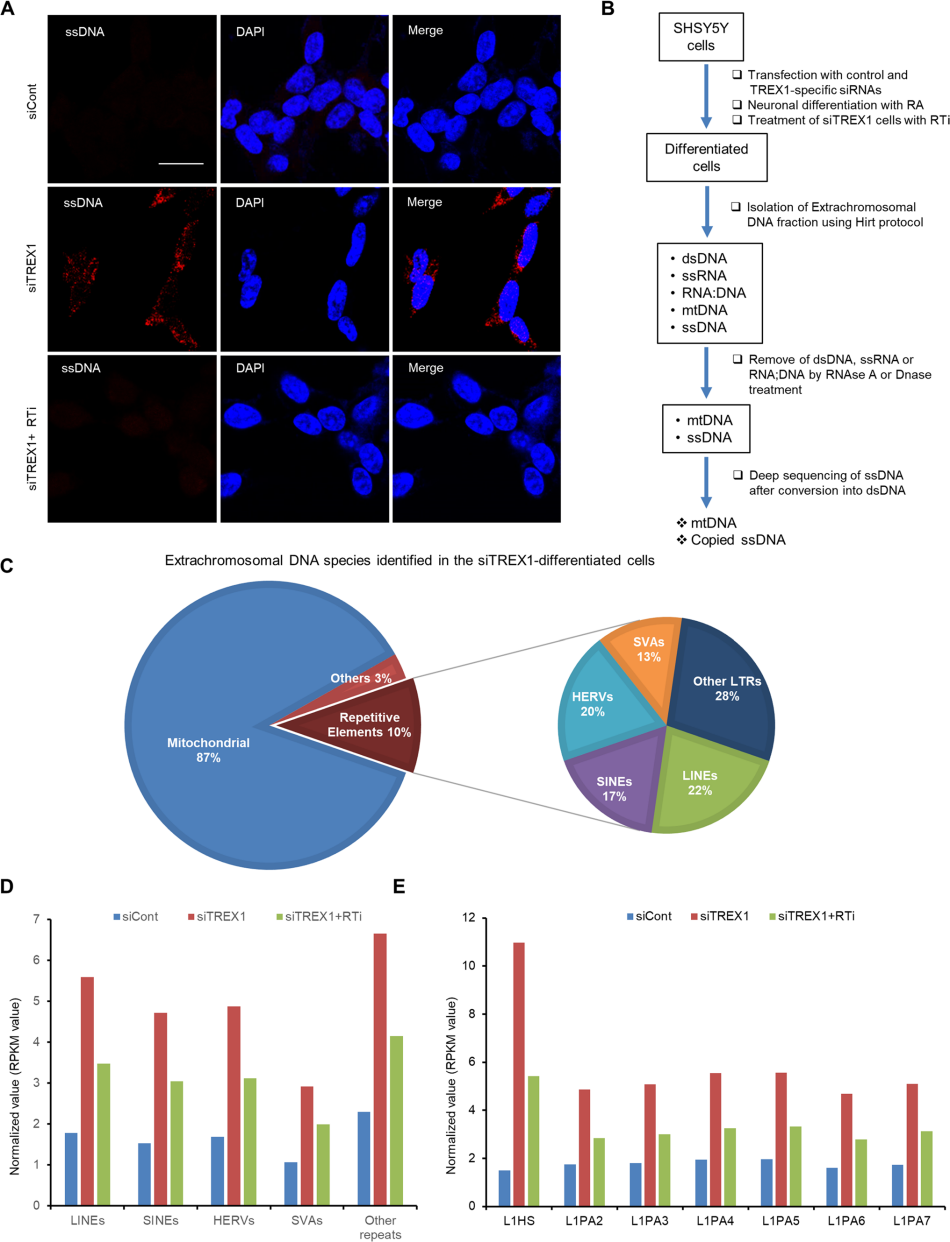
TREX1 knockdown results in the accumulation of reverse-transcribed extranuclear DNA species in neuronal cells. SH-SY5Y cells transfected with siCont and siTREX1 were allowed to undergo neuronal differentiation as described above. Where indicated, the siTREX1-transfected cells were chronically treated with a RTi. **A** The accumulation of single-stranded DNA (ssDNA) in TREX1 knockdown cells was determined by immunofluorescence with an anti-ssDNA-specific antibody. Scale bar: 20 µm. **B** Schematic of the protocol used in the current study to extract extrachromosomal ssDNA for deep sequencing. The extrachromosomal DNA was extracted, and the DNA species were characterized as described in the “Materials and Methods” section. **C** Representative graphs depicting the species identified in the extrachromosomal fraction of the differentiated SH-SY5Y cells by deep sequencing. The siCont, siTREX1, and siTREX1 + RTi extrachromosomal fractions were sequenced. The results from the siTREX1 cells are shown. **D** The composition of the DNA species (repetitive elements/ssDNA) in the extrachromosomal fractions determined by deep sequencing. **E** The composition of the LINE (L1) subfamilies in the extrachromosomal fraction

Since the major role of TREX1 is to degrade ssDNA in the cytosol, we examined ssDNA accumulation in TREX1 knockdown cells. The results showed that TREX1-silenced neuronal cells exhibited higher levels of ssDNA than control neuronal cells, and these increased levels could be restored to levels similar to the control levels by treatment with a RTi (Fig. 2A). Moreover, deep sequencing of TREX1 knockdown neuronal cells revealed that TREX1 silencing resulted in an increased number of retroelements, especially LINEs (also known as L1s) and their subfamily species, compared with control cells (Fig. 2B–E). Collectively, these results suggest that in TREX1-deficient neuronal cells, the accumulated ssDNA species may dysregulate the neuronal development process.

### Loss of TREX1 Activates ER Stress and the UPR Pathway in Neuronal Cells

Considering the localization of TREX1 to the ER, we next performed quantitative RT-PCR analysis to examine the activation of UPR signaling genes in TREX1 knockdown SH-SY5Y cells. The results showed that the expression levels of multiple UPR genes, such as Calnexin, BiP/GRP78, PERK, ATF6α, XBP1, and CHOP, were markedly elevated in the TREX1 knockdown cells compared to the control cells, indicating the activation of the ER stress pathway by TREX1 deficiency (Fig. 1H and 1I). Similar observations were obtained from a time course analysis of TREX1 knockdown SH-SY5Y cells and A431 cells (Supplementary Fig. 1A–D). Moreover, the activation of UPR proteins was observed in an immunocytochemical analysis of TREX1 knockdown cells (Supplementary Fig. 1E). However, modulation of TREX1 silencing by the overexpression of TREX1 (pN3-TREX1) reversed the effects of the activated UPR and restored the cellular phenotypes to be nearly identical those observed in control cells (Fig. 1H and 1I; right panel/lane). Although western blot analysis revealed an unusual endogenous TREX1 band upon TREX1 overexpression, we assumed that this band might have been affected by exogenous TREX1 in the plasmid (Supplementary Fig. 1F). In addition, we investigated whether activated ER stress signaling in TREX1-deficient cells occurs through the STING pathway, a known pathway associated with TREX1-deficient autoimmune diseases. Our results show that while STING knockdown significantly abolished the induction of interferon (IFN) genes in TREX1-deficient cells, the activated ER stress signaling genes were not affected, indicating that the activation of ER stress and the UPR in TREX1-deficient cells is independent of the STING-IFN pathway (Supplementary Fig. 1G and 1H). Taken together, these results indicate that TREX1 silencing induces ER stress and UPR signaling in neuronal cells, which may lead to apoptosis.

### The Loss of TREX1 Disrupts ER Ca^2+^ Homeostasis Through the CHOP-ERO1α-IP3R Pathway

**Fig. 3:**
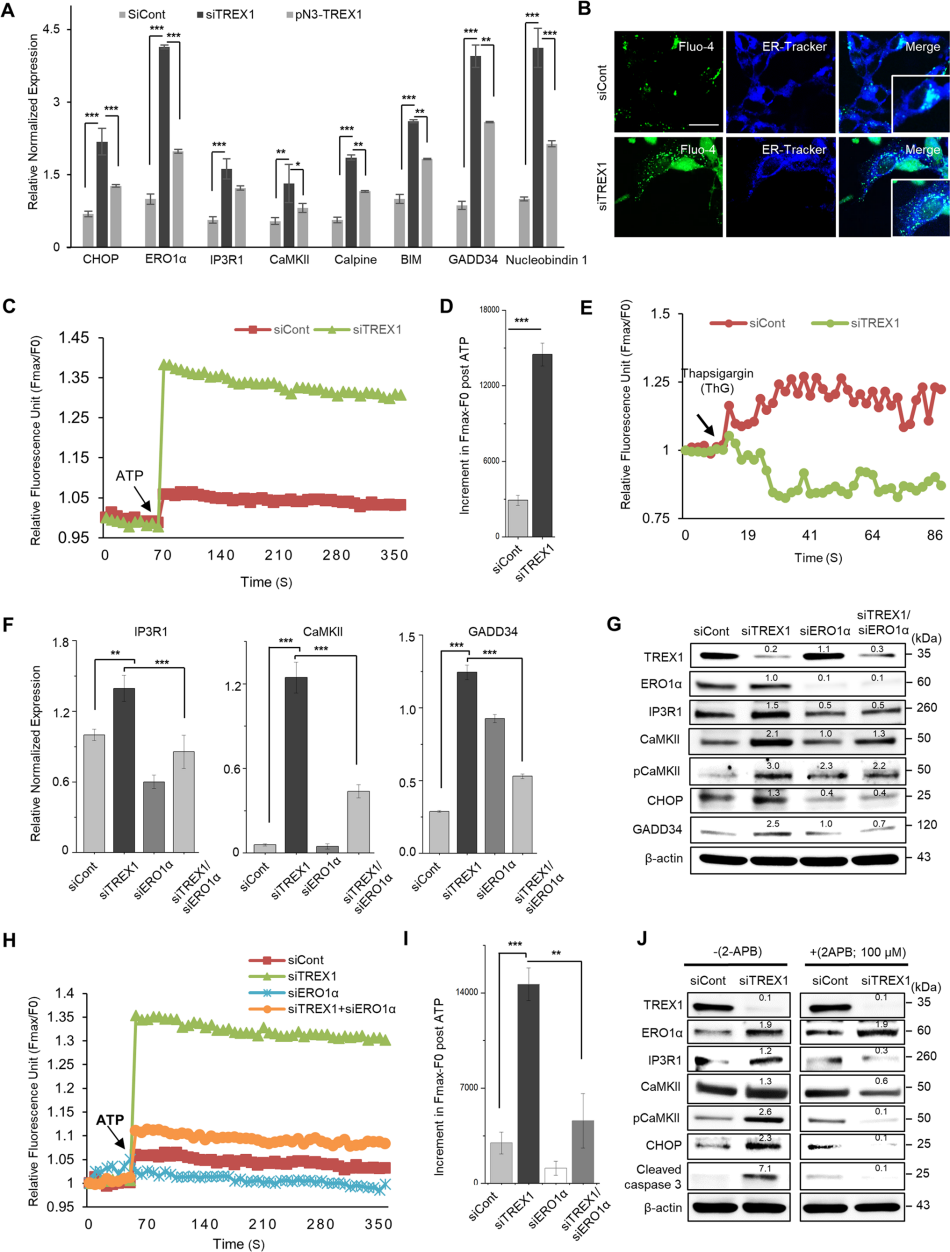
TREX1 deficiency dysregulates ER Ca^2+^ homeostasis via the activation of the CHOP-ERO1α-IP3R1 pathway. SH-SY5Y cells were transfected with siCont and siTREX1 and allowed to undergo neuronal differentiation according to the abovementioned protocol. Where indicated, the effects of TREX1 silencing were reversed by treating siTREX1 cells with the wild-type TREX1 plasmid (pN3-TREX1). **A** The mRNA levels of CHOP and its downstream ER Ca^2+^ regulatory pathway genes were measured by quantitative RT-PCR. **B** The intracellular Ca^2+^ levels were visualized with the Ca^2+^ indicator Fluo-4, and the images were captured by confocal microscopy. The cells were stained ER-Tracker Blue-White DPX. Scale bar: 20 µm. **C** The intracellular Ca^2+^ levels were measured by a spectrofluorometric detection system. The arrow indicates the addition of ATP (10 μM). **D** The bar graph shows the post-ATP increase in the area of Fmax-F0 for the first peak. **E** Thapsigargin-induced Ca^2+^ release from the ER in TREX1 knockdown cells was determined by using a FlexStation detection system. **F**–**I** The effects of silencing ERO1α on the TREX1-mediated activation of IP3R1 and the subsequent Ca^2+^ release from the ER. The mRNA and protein levels of Ca^2+^ signaling targets were measured by quantitative RT-PCR (**F**) and western blot analyses (**G**), respectively. The intracellular Ca^2+^ levels were measured by a spectrofluorometric detection system (**H**). The bar graph shows the post-ATP increase in the area of Fmax-F0 for the first peak (**I**). **J** The protein levels of ER Ca^2+^ regulatory pathway genes were examined in TREX1 knockdown cells treated with an IP3R channel blocker (2-APB, 2 μM) by western blotting. The error bars show the SEM. *, *p* < 0.05; **, *p* < 0.01; ***, *p* < 0.001; One-way ANOVA was performed for multigroup comparisons. The data shown in all the panels are representative of three independent experiments

CHOP is a potent inducer of ER stress-mediated apoptosis, and therefore, we examined CHOP target genes in TREX1 knockdown cells by quantitative RT-PCR and western blot analysis. The results show that TREX1 silencing significantly induces the expression of CHOP target genes, including ERO1α, IP3R1 (not IP3R2 & IP3R3), and CaMKII, which are known regulators of ER Ca^2+^ homeostasis [24–26], indicating that ER Ca^2+^ homeostasis was disrupted in TREX1 knockdown cells (Fig. 3A, Supplementary Fig. 2A–2B and 3A–3B). We next measured the intracellular Ca^2+^ levels in TREX1 knockdown cells using fluorescent (Fluo4-AM) labeling and subsequently detected by confocal imaging or a real-time kinetic detection system. The data revealed that TREX1 silencing markedly increased the intracellular Ca^2+^ levels compared to those in control cells (Fig. 3B–D). In addition, our results show that, compared with control cells, the thapsigargin (ThG)-induced release of ER luminal Ca^2+^ was decreased in TREX1 knockdown cells (Fig. 3E), indicating that the increase in intracellular Ca^2+^ was due to the depletion of ER Ca^2+^. Because the transcriptional activation of CHOP targets is involved in the regulation of ER Ca^2+^ homeostasis, our results suggest that the increased intracellular Ca^2+^ levels in TREX1-silenced cells may be due to the activation of CHOP targets, especially ERO1α.

In cells undergoing ER stress, ERO1α activates IP3R and releases Ca^2+^ from the ER, which in turn activates the Ca^2+^‑sensing kinase CaMKII and thereby triggers apoptosis [24–26]. We found that tunicamycin (an ER stress-inducing agent) led neither to the activation of IP3R1 and CaMKII nor to the elevation in the intracellular Ca^2+^ levels in ERO1α-silenced cells, compared to siCont cells treated with tunicamycin (Supplementary Fig. 2C–E), indicating the triggering of an ERO1α-dependent Ca^2+^ response process under ER stress conditions. Next, we explored the involvement of ERO1α in the disruption of ER Ca^2+^ homeostasis in TREX1-deficient cells. The results showed that while activation of the genes in the ERO1α-IP3R pathway was observed in the TREX1 knockdown cells, ERO1α silencing abolished their activation in the TREX1 knockdown cells (Fig. 3F, 3G and Supplementary Fig. 2F). Moreover, ERO1α silencing diminished the elevated levels of intracellular Ca^2+^ in the TREX1 knockdown cells to be similar to the levels of the siCont cells (Fig. 3H and 3I). In addition, we found that the IP3R antagonist 2-aminoethoxydiphenylborate (2-APB) reduced the expression of IP3R1 and CaMKII without affecting ERO1α in TREX1-silenced cells compared to untreated cells (Fig. 3J and Supplementary Fig. 2G). Together, these data indicate that dysregulated ER Ca^2+^ homoeostasis in TREX1 knockdown cells is mediated through the activation of the CHOP-ERO1α-IP3R1 pathway.

### The Interaction Between TREX1 and BiP/GRP78 Interferes with ER Stress Signaling

**Fig. 4:**
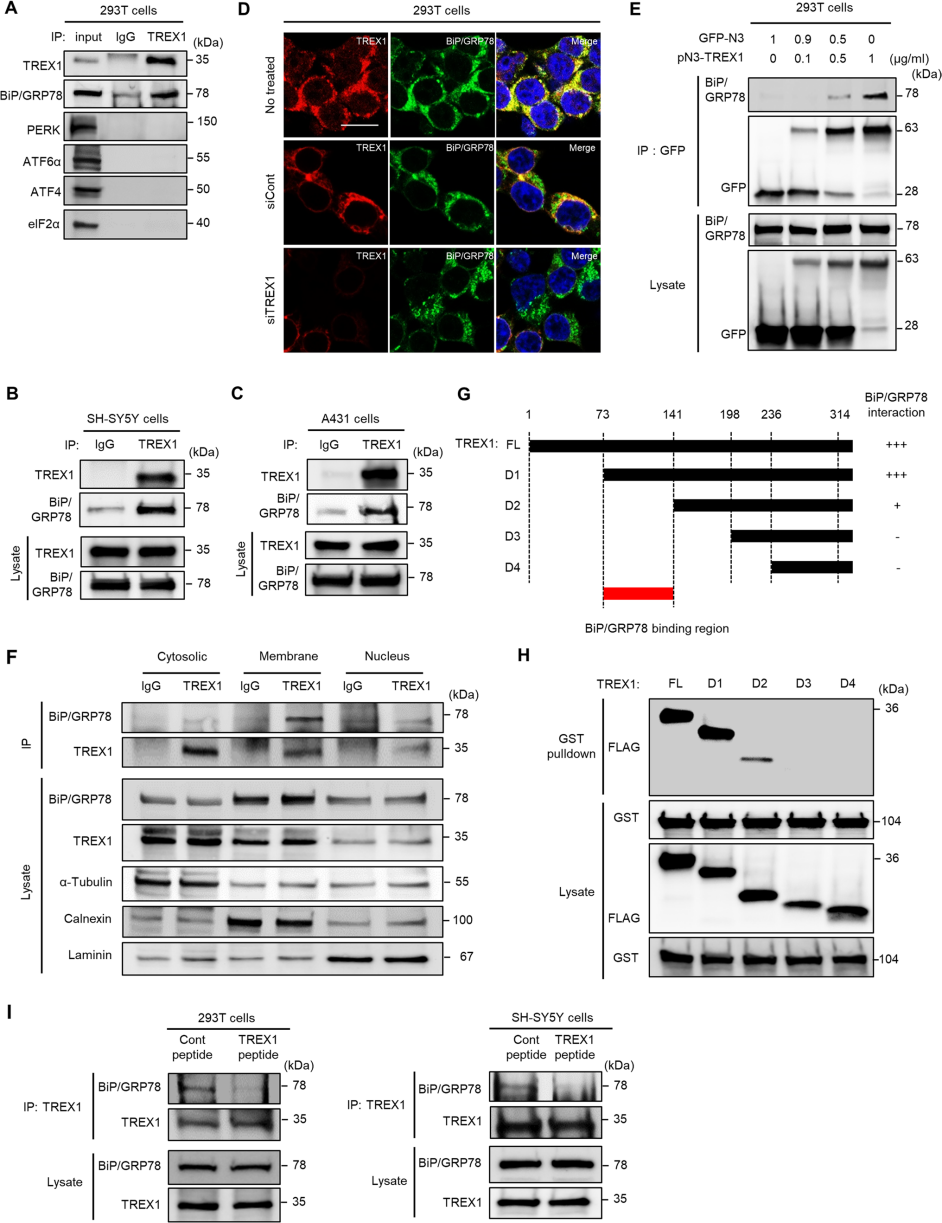
TREX1 interacts with BiP/GRP78. **A**–**C** The interaction between TREX1 and BiP/GRP78 in 293 T, SH-SY5Y, and A431 cells was determined by using an immunoprecipitation (IP) assay. **D** Immunocytochemical analysis of endogenous TREX1 and BiP/GRP78 proteins in 293 T cells. Yellow indicates the colocalization of TREX1 and BiP/GRP78. **E** The specific dose-dependent interaction between TREX1 and BiP/GRP78 was confirmed in 293 T cells transfected with GFP-tagged TREX1 plasmid (pN3-TREX1). **F** The interaction between TREX1 and BiP/GRP78 was detected in membrane fractions containing ER. **G** To dissect the BiP/GRP78-specific binding region of TREX1, full-length (FL) and four deletion constructs encoding amino acids 73–314 [D1], 141–314 [D2], 198–314 [D3], and 236–314 [D4] of TREX1 were cloned into a p3XFLAG-CMV-7.1 vector. **H** The protein–protein interactions between the TREX1-specific regions (73–141) and BiP/GRP78 were investigated using a GST pull-down assay. **I** IP assay of the TREX1-BiP/GRP78 interaction in cells treated with the TREX1 peptide (5 μM), which represents the regions of TREX1 spanning amino acids 80–99

To determine how TREX1 deficiency activates UPR signaling, we examined TREX1 target proteins by immunoprecipitation (IP) assay. The IP analysis revealed that among several candidate ER stress proteins, only BiP/GRP78 clearly interacted with TREX1 in 293 T, SH-SY5Y, and A431 cells (Fig. 4A–C). Immunocytochemical analysis also showed the strong colocalization of TREX1 and BiP/GRP78 in these cells (Fig. 4D and Supplementary Fig. 4A). In addition, a strong concentration-dependent interaction between overexpressed GFP-tagged TREX1 and endogenous BiP/GRP78 was evident (Fig. 4E). This interaction was confirmed in membrane fractions containing ER (Fig. 4F). Furthermore, TREX1 interacts with BiP under non-ER stress condition and their interaction decreased under ER stress conditions (Supplementary Fig. 4B).

To investigate the binding region of TREX1 with BiP/GRP78, we constructed deletion fragments (D1-D4) of TREX1 and assessed the interaction by IP. Remarkably, the results showed that the region of the TREX1 protein containing amino acids 73–141 was involved in BiP/GRP78 binding (Fig. 4G and 4H). In addition, the IP analysis with a TAT-conjugated TREX1 mimic peptide targeted to the binding region (80–99 amino acids) clearly showed that the peptide impeded the interaction between TREX1 and BiP/GRP78 (Fig. 4I and Supplementary Fig. 4C). As expected, peptide treatment activated the ER stress pathway, which led to apoptosis via the ERO1α-IP3R1-CaMKII pathway (Supplementary Fig. 4D–4F). However, the peptide treatment did not alter the ER localization or nuclear activity of TREX1 (Supplementary Fig. 4G and 4H). Together, these findings indicate that TREX1-deficient cells triggered ER stress and UPR signaling via disruption of the TREX1-BiP/GRP78 interaction.

TREX1 Knockdown Results in Golgi Fragmentation and Impairs Microtubule Acetylation.

**Fig. 5:**
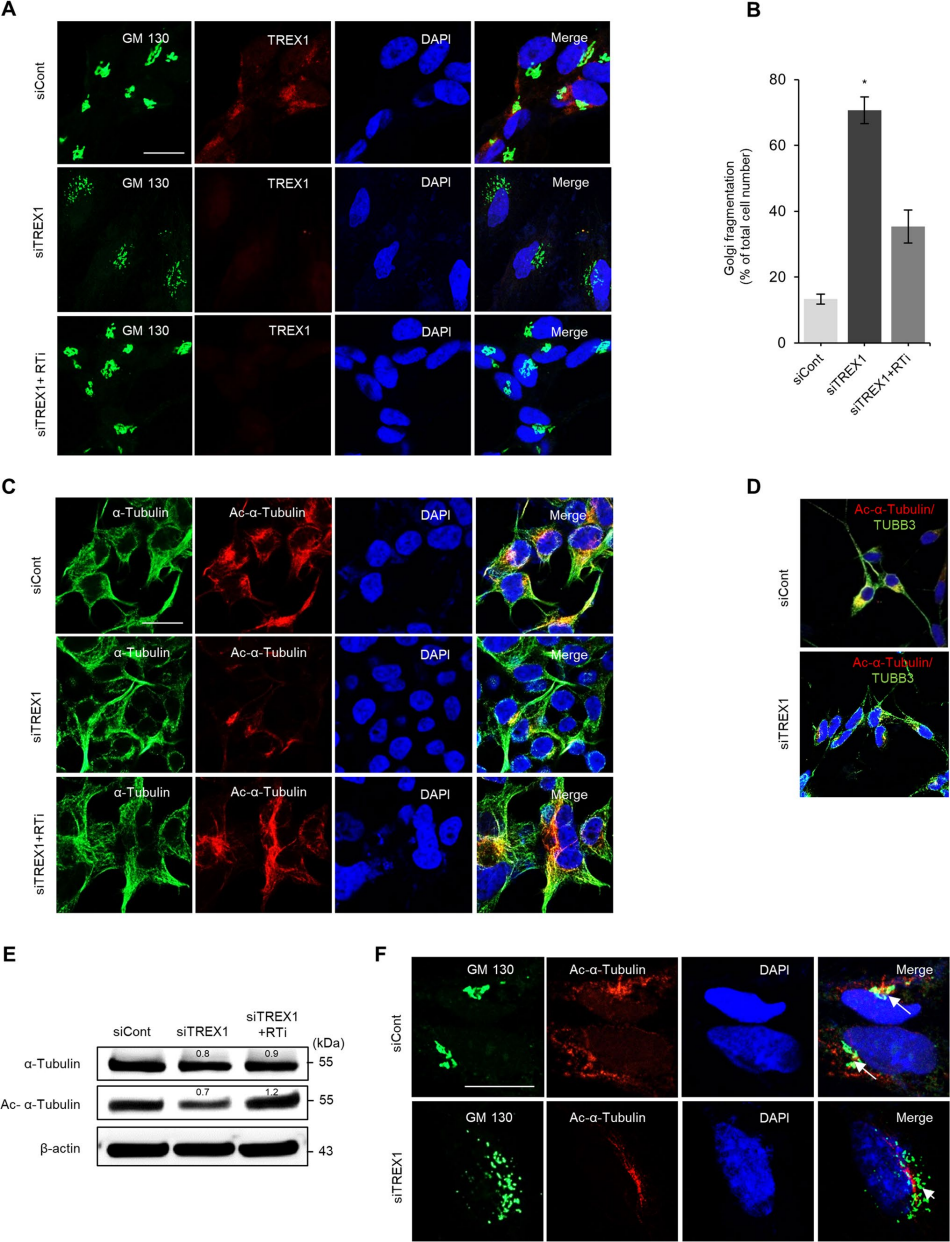
TREX1 deficiency results in increased Golgi fragmentation and reduced microtubule acetylation in neuronal cells. SH-SY5Y cells transfected with siTREX1 and siCont were allowed to undergo differentiation as described above. **A** The loss of TREX1 in neuronal cells promotes Golgi fragmentation. Scale bar: 50 µm. **B** Graphical presentation of the cells with fragmented Golgi. **C** The cells were immunostained with an antibody against α-tubulin (green) and its acetylated form (red). **D** Costaining of TUBB3 (green) and Ac-α-tubulin (red). Scale bar: 20 µm. **E** Protein levels of α-tubulin and its acetylated form. **F** TREX1 knockdown results in decreased microtubule acetylation coupled with reorganization of the Golgi complex. In control cells, cis-Golgi (GM 130) tightly associates with Ac-α-tubulin, whereas TREX1 deficiency impairs the tight association of the Golgi with tubulin (arrows vs. arrowheads). Scale bar: 20 µm. The error bars show the SEM (*n* = 3). *, *p* < 0.05

The Golgi is an important cellular organelle that plays important roles in the maintenance and function of neuronal cells, and fragmentation of the Golgi is commonly observed in neurodegenerative cells subjected to ER stress [27, 28]. Immunocytochemical analysis showed that TREX1 silencing resulted in fragmentation of the Golgi, as shown by increased punctate-like formation of GM-130 (a cis-Golgi marker) in TREX1 knockdown cells; this phenomenon was negligible in control cells (Fig. 5A and 5B). In addition, TREX1 deficiency disrupted microtubule stability in neuronal cells, as shown by the markedly reduced level of Ac-α-tubulin in TREX1 knockdown cells compared to control cells (Fig. 5C–E). Furthermore, since the spatiotemporal association of Ac-α-tubulin and cis-Golgi is has been established [29, 30], we analyzed the association of Ac-α-tubulin and cis-Golgi in TREX1 knockdown cells. The results revealed that, compared with the control cells, TREX1-silenced neuronal cells exhibited decreased microtubule acetylation coupled with reorganization or fragmentation of the cis-Golgi (Fig. 5F; arrows show control cells and arrowheads show TREX1-silenced cells). Together, our observations suggest that TREX1 silencing may disrupt intracellular trafficking involving the Golgi, which in turn results in the impairment of neuronal homeostasis and degeneration.

### The V91M Mutation in TREX1 Induces ER Stress-Mediated Ca^2+^ Release and Golgi Fragmentation, Leading to Neurodegeneration

**Fig. 6:**
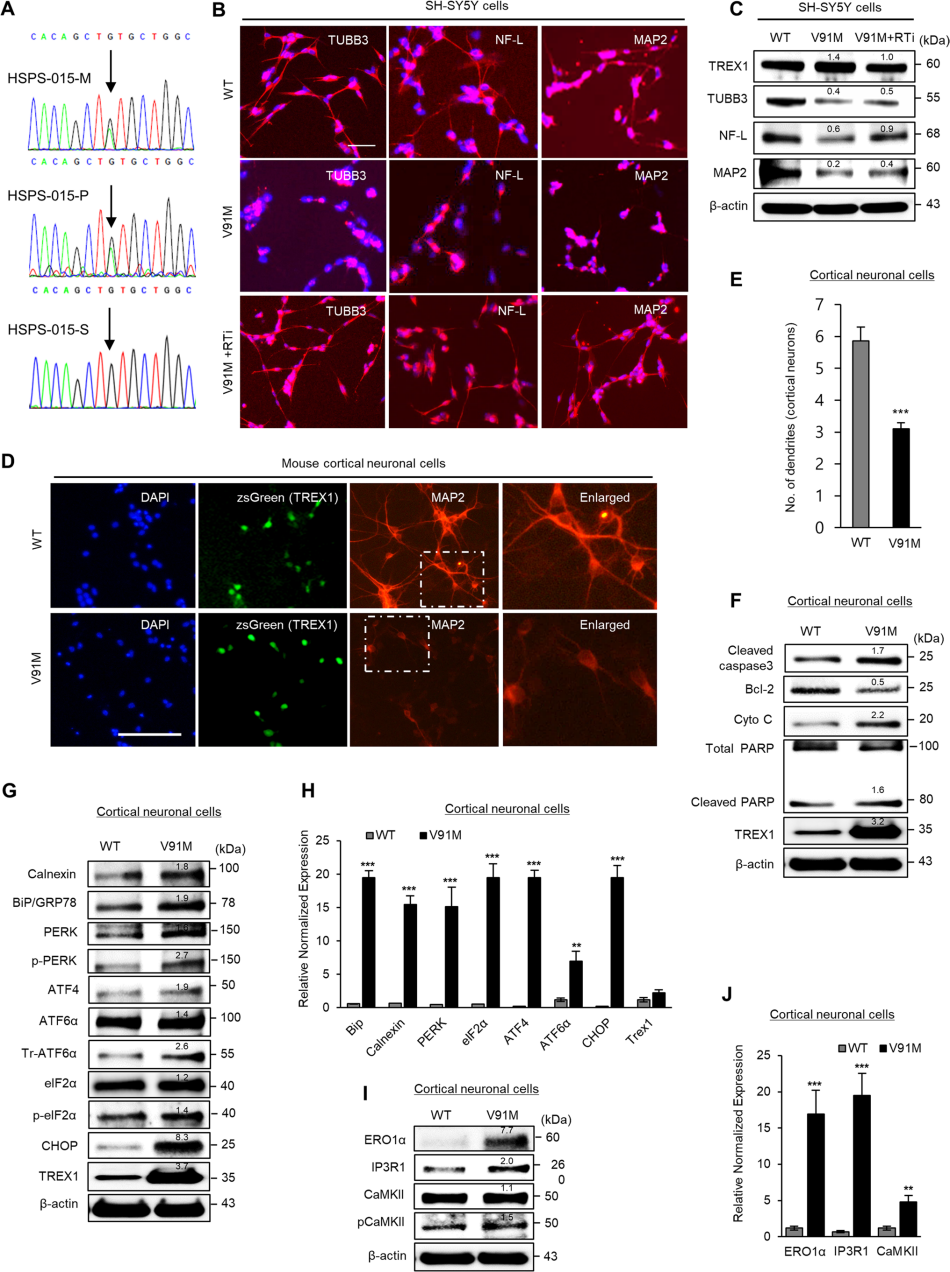
The HSP patient-specific mutation in TREX1 (V91M) impairs neuronal development, induces ER stress and the UPR, and causes apoptosis. **A** Representative Sanger sequence traces of the TREX1 mutation that was identified in Korean HSP patients and normal individuals. M, mother (born in 1965); P, proband (born in 1992); and S, sibling (born in 1994). SH-SY5Y cells or mouse primary cortical neurons were transfected with plasmids expressing mutant (V91M) and wild-type TREX1 and allowed to undergo differentiation. Where indicated, the effects of the TREX1 mutation were reversed by treating TREX1-mutant cells with reverse transcriptase inhibitors. **B**, **C** The expression levels of neuron-specific markers in differentiated neuronal cells were determined by immunocytochemistry (**B**) and western blotting (**C**). Scale bar: 50 µm. **D** The effects of the Trex1 mutant on primary neuronal cells were evaluated by immunostaining with the MAP2 antibody. **E** Graphical presentation of the number of dendrite outgrowths. **F** Western blot analysis of apoptotic proteins in primary neuronal cells. **G** The expression level of UPR pathway proteins in primary cortical neurons transfected with plasmids expressing mutant (V91M) and wild-type TREX1. **H** The mRNA level of UPR pathway genes in primary cortical neurons. The protein (**I**) and mRNA (**J**) levels of Ca^2+^ pathway genes in primary cortical neurons

We analyzed our whole-exome sequencing (WES) data from 87 Korean families with HSP, which is a representative neurodegenerative disorder. We identified the pathogenic c.271G > A missense mutation (heterozygous) in exon 2 of the TREX1 gene in one Korean family with HSP showing an autosomal dominant inheritance (Fig. 6A and Supplementary Fig. 5A); in this family, the patient and his mother were diagnosed with HSP with insidious onset of lower extremity spasticity and weakness, brain atrophy, and white matter change (Supplementary Fig. 5B and Supplementary Table 1). The c.271G > A mutation results in an amino acid substitution of valine to methionine at position 91 (NM_033629.6: exon 2: c. G271A: p.V91M).

To address the effect of this TREX1 mutation on neuronal development, we constructed a plasmid and lentiviral vector expressing TREX1-V91M and then evaluated the neuronal differentiation process in SH-SY5Y cells and mouse primary cortical neuronal cells harboring the plasmid. We observed that the TREX1 mutant significantly impaired the neuronal differentiation process in SH-SY5Y cells (Fig. 6B, 6C and Supplementary Fig. 5C). The TREX1 mutant also significantly affected the morphology of primary cortical neuronal cells, as a significant reduction in axonal outgrowth was observed in the TREX1-mutant cells compared to the wild-type cells (Fig. 6D and 6E). Transfection with the TREX1 mutant also drove mouse cortical neuronal cells to apoptosis, but transfection with wild-type TREX1 did not have this effect (Fig. 6F).

Compared with wild-type TREX1, the TREX1 mutant increased the number of propidium iodide (PI)-positive cells, indicating that the TREX1 mutant affects the survival of primary cortical neuronal cells (Supplementary Fig. 5D and 5E). In addition, our data revealed that the TREX1 mutant caused remarkable cell stress and affected cell viability (Supplementary Fig. 5F–H). Because TREX1-V91M is a heterozygous mutation and due to its autosomal dominant nature, V91M might show a damaging effect in neuronal cells even in the presence of wild-type TREX1. Together, these results indicate that the TREX1 mutation impairs neuronal homeostasis and mediates apoptosis.

**Fig. 7:**
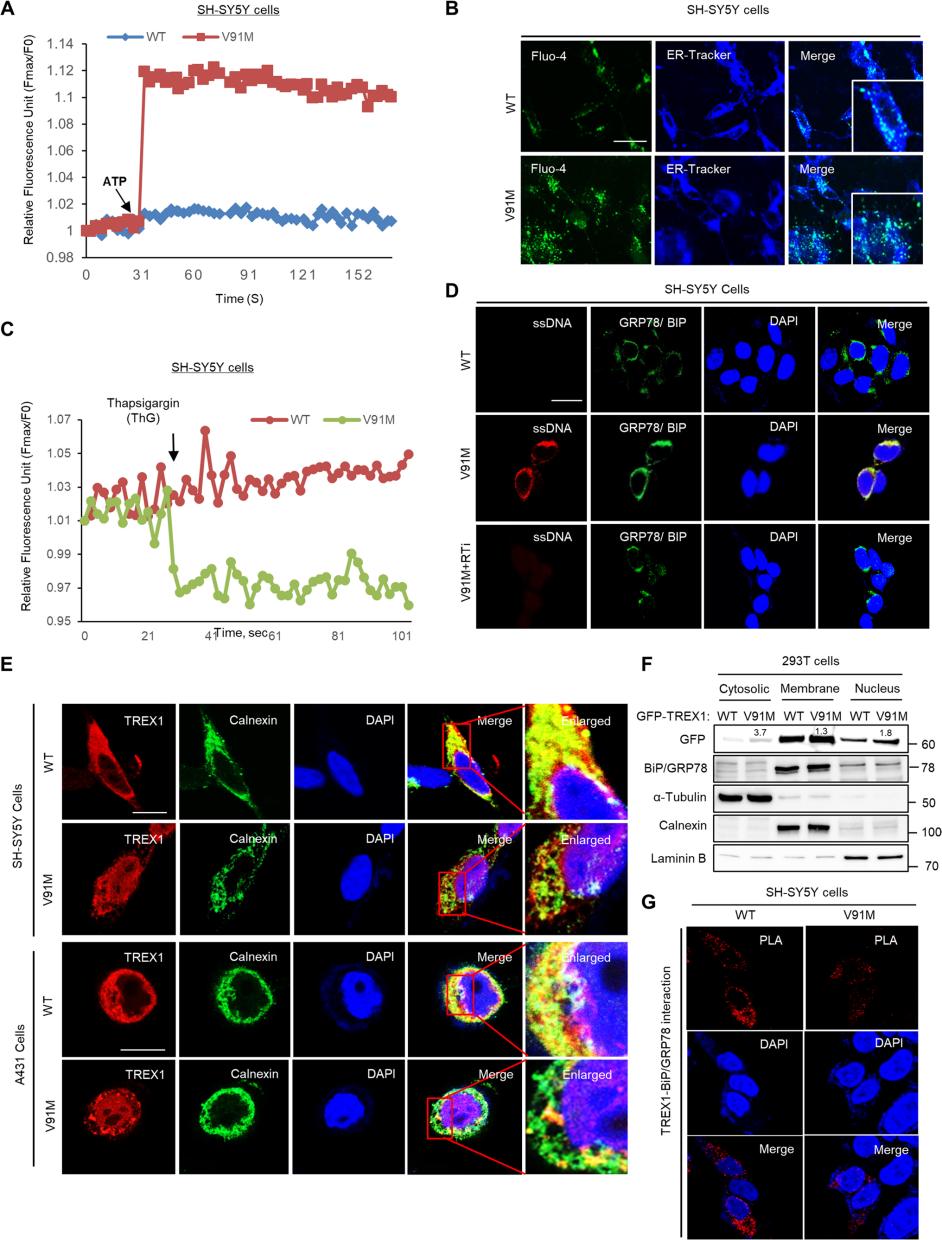
The TREX1 mutant dysregulates ER Ca^2+^ homeostasis and impairs the localization and function of TREX1. **A**–**C** SH-SY5Y cells transfected with plasmids expressing the mutant (V91M) and wild-type TREX1 were allowed to undergo differentiation. The intracellular Ca^2+^ levels in TREX1-mutant SH-SY5Y cells were measured by a spectrofluorometric detection system (**A**) and visualized with Fluo-4 by confocal microscopy (**B**). The cells were stained with Fluo-4 in green and ER-Tracker Blue-White DPX. Scale bar: 20 µm. To assess ER stress-mediated Ca^2+^ release from the ER, the cells were loaded with Fluo-4 and then treated with 2 µM thapsigargin. With a FlexStation, the plate was read before and after the addition of thapsigargin at ~ 2 s intervals for approximately 110 s (**C**). **D** The accumulation of ssDNA in TREX1-mutant plasmid-transfected cells was determined by immunostaining with an antibody against ssDNA (red). Green denotes the ER marker GRP78/BIP. The siTREX1 cells were chronically treated with RTi. **E** The localization of TREX1 in TREX1-mutant SH-SY5Y and A431 cells was determined by immunostaining with antibodies against TREX1 (red) and Calnexin (an ER marker; green). **F** The expression of wild type and point mutant TREX1 in subcellular fractionations. **G** Representative images of proximity ligation assays (PLA) and DAPI in SH-SY5Y cells transfected with TREX1 WT and V91M. The error bars show the SEM. Scale bar: 20 µm

**Fig. 8:**
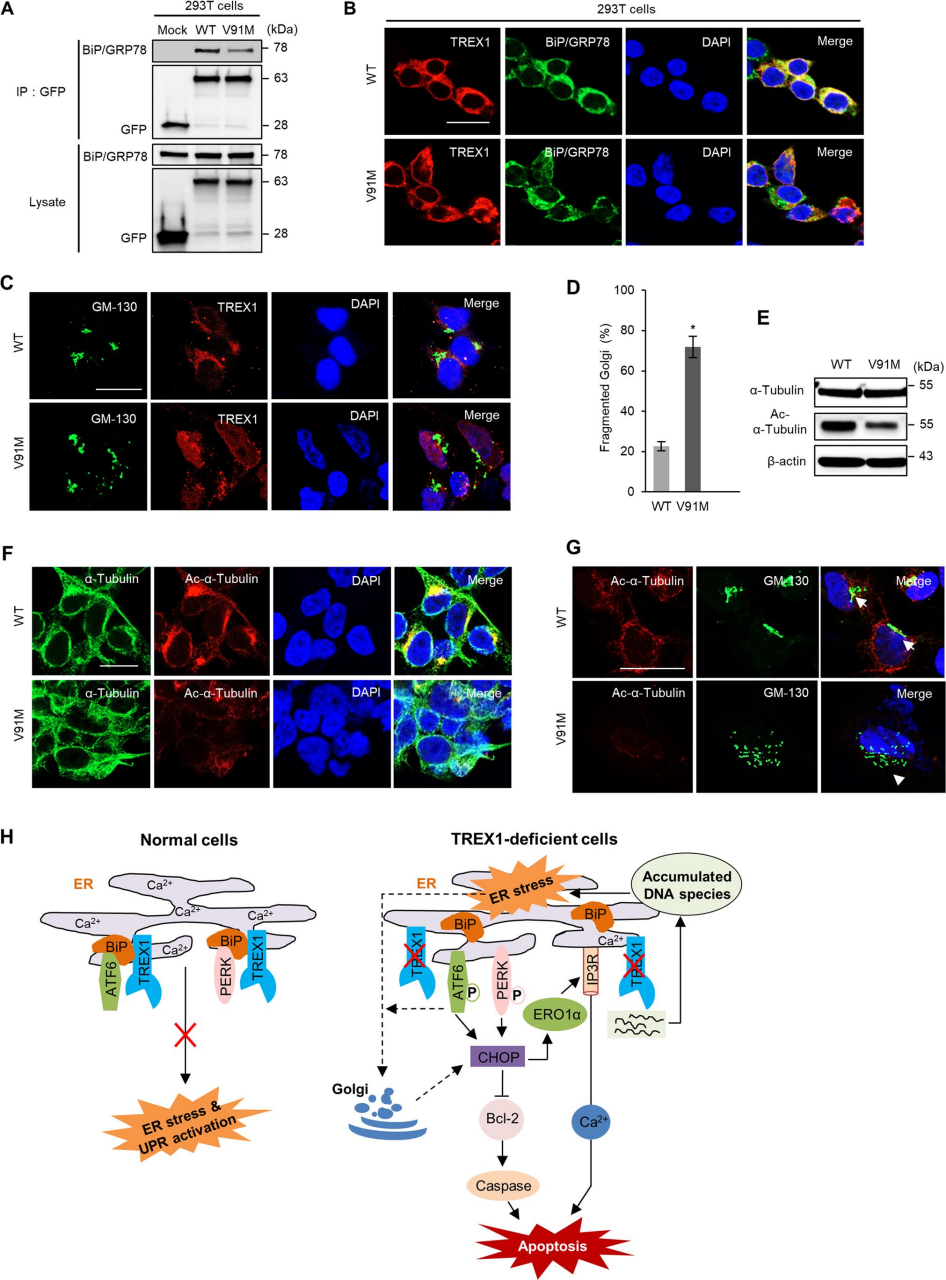
The TREX1 mutant impairs microtubule acetylation and Golgi fragmentation in neuronal cells. **A** An IP assay with anti-GFP antibody was carried out with 293 T cells transfected with GFP-tagged wild-type and mutant TREX1 plasmids (TREX1 missense V91M mutant plasmid). **B** Immunocytochemical analysis showing decreased colocalization (yellow) of TREX1 and BiP/GRP78 in TREX1-mutant 293 T cells. **C**–**G** SH-SY5Y cells were transfected with plasmids expressing mutant (V91M) and wild-type TREX1 and then allowed to undergo neuronal differentiation. Golgi fragmentation was determined by immunostaining with antibodies against cis-Golgi (GM-130; green) and TREX1 (red) (C). Scale bar: 50 µm. Graphical representation of Golgi-fragmented cells (**D**). The expression level of α-tubulin and its acetylated form were analyzed by western blotting (**E**) and immunocytochemistry (**F**). TREX1 mutation results in decreased microtubule acetylation coupled with reorganization of the Golgi complex (**G**). In wild-type cells, cis-Golgi (GM 130) tightly associated with Ac-α-tubulin, whereas the TREX1 missense mutation impaired the tight association of the Golgi with tubulin (arrows vs. arrowheads). Scale bar: 20 µm. The error bars show the SEM (*n* = 3). *, *p* < 0.05. The data shown in all the panels are representative of three independent experiments. **H** Schematic representation of the mechanisms involved in neuronal cell death due to TREX1 deficiency

We found that the TREX1 mutant activated ER stress and UPR signaling in both primary cortical and differentiated neuronal cells (Fig. 6G and 6H and Supplementary Fig. 5I–5 J). In addition, the TREX1 mutant caused ER stress-induced CHOP activation, which in turn activated the ERO1α-IP3R1-CaMKII pathway in neuronal cells (Fig. 6I and 6J and Supplementary Fig. 5 K). In line with these observations, the TREX1 mutant disrupted ER Ca^2+^ homeostasis, leading to Ca^2+^ leakage from the ER and thereby increasing the intracellular Ca^2+^ levels in TREX1-mutant cells compared to those in wild-type cells (Fig. 7A–C). Surprisingly, the TREX1 mutant was mislocated away from the ER as shown in data of confocal microscopy and fractionation analysis, which subsequently allowed the accumulation of ssDNA in the ER (Fig. 7D–7F). Moreover, the TREX1 mutant showed weak binding and diminished colocalization with BiP/GRP78 compared to wild-type TREX1 (Figs. 7G and 8A–B and Supplementary Fig. 5L).

Our data revealed that the TREX1 mutant caused fragmentation of the Golgi, as shown by the increase in punctate-like formations of GM-130 in the TREX1-mutant cells (Fig. 8C and 8D). Furthermore, the TREX1 mutant resulted in instability of the Golgi-microtubule network in neuronal cells, as shown by the reduction in microtubule acetylation and the increase in Golgi fragmentation in the TREX1-mutant cells compared to the wild-type cells (Fig. 8E–G). Collectively, our data suggest that a missense mutation (V91M) in TREX1 causes the activation of ER stress and the UPR, which in turn results in neurodegeneration through the disruption of ER Ca^2+^ homeostasis and/or the Golgi-microtubule network and thereby contributes to the progression of HSP.

## Discussion

Herein, we described a newly discovered mechanism of TREX1 function that was discovered through the activation of ER stress and UPR signaling in response to TREX1 deficiency, which led to neuronal cell apoptosis (Fig. 8H). Importantly, we confirmed, for the first time, that a missense mutation in the TREX1 gene, which was identified in a Korean family with HSP, caused neurodegeneration through a novel mechanism, indicating that TREX1 deficiency contributes to the progression of HSP.

In this study, we demonstrate that the aberrant accumulation of cytosolic DNA species due to TREX1 depletion impaired neuronal homeostasis and increased neuronal cell dysfunction. In addition, our data reveal that in TREX1-deficient cells, reverse-transcribed ssDNA species accumulated in the ER, which might have resulted from altered exonuclease activity and the mislocalization of TREX1 away from the ER, leading to impairment in neuronal function. These findings suggest that the proper function and localization of TREX1 in the ER are important, a supposition supported by previous findings showing that TREX1 mislocalization away from the ER results in the accumulation of ssDNA, which ultimately induces cell death [1, 31]. Importantly, our data demonstrated that TREX1 clearly interacted with the ER chaperone BiP/GRP78, while TREX1 depletion impaired TREX1-BiP/GRP78 binding. A region containing 73–141 amino acids of the TREX1 protein was found to be involved in the interaction with BiP/GRP78. Under normal conditions, BiP/GRP78 is known to bind three ER stress transducers, PERK, IRE1α, and ATF6α, repressing their activation by maintaining their inactive monomeric states, and dissociation of BiP/GRP78 during ER stress is correlated with PERK, IRE1α, and ATF6α activation [12]. Our data revealed that treatment with a TREX1 mimic peptide or the induction of TREX1 deficiency led to the impairment of TREX1 and BiP/GRP78 binding, which subsequently activated UPR sensors. These results suggest that the TREX1-BiP/GRP78 interaction may be involved in the stability of BiP/GRP78-PERK and BiP/GRP78-ATF6α binding, whereas TREX1 depletion results in the dissociation of PERK/ATF6α from BiP/GRP78, leading to the activation of UPR sensors. In addition, activated ER stress signaling in TREX1-deficient cells was shown to be independent of the STING-IFN pathway, which is compatible with previous studies showing that STING-mediated ER stress activation is independent of the interferon regulatory factor 3 (IRF3)-IFN signaling axis [32]. Taken together, these results suggest that disruption of the TREX1-BiP/GRP78 interaction might contribute to the activation of UPR signaling in TREX1-deficient cells.

Among the UPR signaling targets, TREX1-deficient cells contain activated CHOP, which is known to play important roles in ER Ca^2+^ homeostasis and apoptosis. We demonstrated that TREX1 deficiency induces the activation of CaMKII, which is a key signaling event that links CHOP-induced Ca^2+^ release from the ER to apoptosis [25]. Mechanistically, we show that TREX1 deficiency causes CHOP-mediated activation of ERO1α, which subsequently activates IP3R1 and releases Ca^2+^ from the ER. These results are supported by previous studies showing that CHOP-induced ERO1α activates the IP3R receptor, leading to Ca^2+^ release from the ER, which may contribute to apoptosis [25, 33]. In addition, TREX1 silencing increased the expression of the purinergic receptor P2Y1, which is known to trigger the activation of IP3R1 and sensitize cells to ER Ca2 + drainage, and thus, it may have also increased intracellular Ca2 + levels in TREX1 knockdown cells (Supplementary Fig. 3C and 3D). Although the molecular details of luminal redox regulation by IP3R remain to be determined, our findings suggest an interesting function of TREX1 in the maintenance of Ca^2+^ homeostasis via the ERO1α-IP3R1-CaMKII pathway, which appears to be an important function for neuronal cell survival. Furthermore, altered TREX1 function disrupts the stability of microtubules and results in fragmentation of the Golgi. Fragmentation of the Golgi might be a consequence of altered TREX1 on the UPR signaling protein ATF6α and/or the disruption of ER Ca^2+^ homeostasis in neuronal cells [34].

Importantly, our data provide evidence that mutation in the ER-associated gene TREX1 is the likely cause of HSP in a Korean family. We demonstrated that a missense mutation in the TREX1 gene resulted in ER stress-mediated neurodegeneration in both differentiated and mouse primary cortical neuronal cells. This deleterious change is due to ER stress-induced Ca^2+^ release via the ERO1α-IP3R1-CaMKII pathway. The observations of ER Ca^2+^ homeostasis disruption and HSP development as a result of altered TREX1 function are supported by recent studies on the pathogenesis of HSP, which showed that mutations in ER-related genes and their associated Ca^2+^ homeostasis pathways are extensively involved in the progression of HSP [16, 18, 35, 36]. Importantly, TREX1-mutant neuronal cells showed fragmented Golgi and/or disorganized microtubules, outcomes that have also been reported in HSP disease [18, 21, 22]. Golgi fragmentation prior to apoptosis is often detected as an early event in pathological conditions [34, 37], suggesting that the disrupted Golgi-microtubule network in TREX1-mutant neuronal cells can trigger neurodegeneration and thus contribute to the progression of HSP. While an in vitro condition does not fully recapitulate the complexity of HSP disease, further studies with mouse models of TREX1 mutation would be required to dissect HSP disease progression caused by TREX1-deficient functions.

TREX1 deficiency leads to cytosolic ssDNA accumulation, oxidative stress-related inflammation, and subsequent cell death [1]. Heterozygous mutations of TREX1 have been identified in patients with autoimmune disease such as systemic lupus erythematosus and Sjögren’s syndrome [38]. Besides, it has been reported that the anti-inflammatory reaction through redox status regulation provides neuroprotection. In contrast, failure to alleviate the stress through an adequate anti-inflammatory response causes neurodegeneration and neuronal cell death [39, 40]. Thus, we demonstrated the transcriptional changes in interferons in TREX1-deficient cells (Supplementary Fig. 1G), supporting the relevance of TREX1 function in inflammatory response pathways. These findings suggest that the expression change in inflammatory response pathways involving vitagenes, including antioxidant genes, are a pathogenic cause of HSP. Therefore, the interplay of ER Ca2 + homeostasis disruption and inflammatory response pathways is worth exploring for further understanding of the pathogenic cause of HSP in TREX1-mutation-bearing patients.

In summary, the current study demonstrates a newly discovered mechanism by which TREX1 acts as an important ER stress regulator through its association with the ER chaperone BiP/GRP78 and shows that the altered function of TREX1 induces chronically elevated ER stress and UPR signaling and primes neuronal cells for neurodegeneration and death. Furthermore, we provide evidence that a TREX1 mutation is the likely cause of HSP and reveal the ER Ca^2+^ homeostasis pathway, which has been shown to be impaired in many neurodegenerative diseases, is a candidate mechanistic pathway associated with HSP. Overall, the new mechanistic insights described in this study suggest novel therapeutic strategies targeting the ER stress-mediated Ca^2+^ signaling pathway to prevent TREX1-associated diseases.

## Materials and Methods

### Cell Culture and Differentiation

The human SH-SY5Y neuroblastoma cell line was grown in high-glucose DMEM (with sodium pyruvate and without l-glutamine; HyClone, 30,285) supplemented with 1% nonessential amino acids (NEAA; Gibco; 11,140–050), 1% GlutaMAX supplement (Gibco; 35,050–061), 1% penicillin/streptomycin (Gibco; 15,140), and 10% FBS. The cells were grown to confluence in a humidified atmosphere (5% CO_2_) at 37 °C in 100 cm^2^ tissue culture dishes. The human A431 and 293 T cell lines were grown in high-glucose Dulbecco’s modified Eagle’s medium (DMEM) with l-glutamine and sodium pyruvate (HyClone, Logan, UT, USA; SH30243) supplemented with 10% foetal bovine serum (FBS; Gibco, Gaithersburg, MD, USA; #16,000) and 1% penicillin/streptomycin. The cells were maintained in a humidified atmosphere (5% CO_2_) at 37 °C in 100 cm^2^ tissue culture dishes.

The SH-SY5Y cells were differentiated into neurons by using a previously published protocol with some modifications [41]. The differentiation of SH-SY5Y cells into neurons was carried out in two steps using retinoic acid (RA; Sigma-Aldrich, St. Louis, MO, USA; R2625 and brain-derived neurotrophic factor (BDNF; Sigma-Aldrich; SRP3014, Supplementary Table 2). In the first step, high-glucose DMEM (with sodium pyruvate and without l-glutamine (HyClone; 30,285)) was supplemented with 3% FBS, 1% NEAA, 1% GlutaMAX supplement and 1% penicillin/streptomycin. The medium was further supplemented with all-trans RA (10 μM) before being applied to the cells. After 24 h of RA treatment, half of the medium was replaced with fresh medium including RA. In the second step, after 48–72 h of RA treatment, the cells were incubated with high-glucose DMEM (with sodium pyruvate; without l-glutamine (HyClone; 30,285)) supplemented with 50 ng/mL BDNF, 1% NEAA, 1% GlutaMAX supplement and 1% penicillin/streptomycin but without serum. Fresh BDNF (50 ng/mL) was added shortly before applying the medium to the cells. The differentiated cells were harvested and analyzed.

### Preparation of Mouse Primary Cortical Neurons and Lentiviral Transfection

C57BL/6 mice were maintained in accordance with the Guidelines for Animal Care and Use, KRIBB. The primary cortical neurons were prepared as previously described [42]. The neocortex regions were dissected from C57BL/6 mouse brains at embryonic day 16 and were dissociated into a single-cell suspension using 0.1% trypsin–EDTA solution. The dissociated cells (1.5–3 × 10^5^ cells/well) were plated in Matrigel-coated 24-well plates with culture medium (neurobasal medium (Gibco; 21,103,049) supplemented with B-27 (Gibco; 17,504,044) and 1% GlutaMAX (Gibco; 35,050)). After 24 h, the cells were infected with lentivirus particles containing wild-type Trex1 and patient-derived mutant Trex1 in FBS-free medium. For the generation of the lentiviral constructs, the pLVX-EF1α-IRES-ZsGreen1 vector (Clontech Laboratories, Mountain View, CA) was used.

### siRNA Transfection

Small interfering RNA (siRNA) sequences against TREX1 and ERO1α were purchased from Bioneer, Daejeon, Republic of Korea, and control sequences were purchased from ST Pharm (Seoul, Republic of Korea). Then, 50–100 nM of each siRNA was transfected using the Lipofectamine RNAiMAX reagent (Invitrogen, CA, USA; 13,778,150) following the manufacturer’s instructions. The siRNA sequences were as follows: siCon sense: 5′-AUG AAC GUG AAU UGC UCA ATT-3′, antisense: 5′-UUG AGC AAU UCA CGU UCA UTT-3′; siTREX1 sense: 5′-GACCAAGCCAAGACCAUCU-3′, antisense: 5′-AGAUGGUCUUGGCUUGGUC-3′; and siERO1α sense: 5′-CACUCAAGGAGAGUCAUCU-3′, antisense: 5′-AGAUGACUCUCCUUGAGUG-3′.

### Plasmid Construction and Transfection

The full-length cDNA of human TREX1 was purchased from Korea Human Gene Bank (Daejeon, Republic of Korea). TREX1 was subcloned into the GFP-tagged pEGFP-N3 vector to construct the GFP-tagged plasmids. An HSP patient-specific point mutation was generated in TREX1, and the sequence subcloned into the GFP-tagged pEGFP-N3 vector. The mutation was confirmed by sequencing (Bioneer, Daejeon, Korea). The plasmids were transfected into cells using Lipofectamine 2000 (Invitrogen, CA, USA; 1,668,019) following the manufacturer’s protocol. In rescue experiments, the GFP-tagged TREX1 plasmids were transfected into the cells using FuGENE 6 (Promega, WI, USA; E2691) following the manufacturer’s description.

### Oligonucleotide Transfection

Two micrograms (2 µg) of a random 60-mer oligonucleotide was transfected into SH-SY5Y cells using Lipofectamine 2000 (Invitrogen, CA, USA; 1,668,019) following the manufacturer’s protocol. The cells were fixed at 1 h posttransfection. For the S1 nuclease treatment, 500 U/mL of nuclease was added during RNase treatment with the supplied S1 buffer (Promega M5761). The SH-SY5Y cells transfected with oligonucleotides displayed high amounts of ssDNA puncta, whereas the SH-SY5Y cells transfected with oligonucleotides and subsequently treated with S1 nuclease showed little to no puncta.

### RNA Isolation and qRT-PCR Analysis

The total RNA was obtained from the cells by using the RNeasy Mini Kit (Qiagen, CA, USA) following the manufacturer’s instructions. Next, 1 µg of the extracted RNA was reverse transcribed into cDNA using the AccuPower RT PreMix (Bioneer, Daejeon, Republic of Korea). The resulting cDNAs were used to analyze the genes of interest by quantitative RT-PCR with the Bio-Rad PCR system (iQ SYBR^R^ Green Supermix). The reactions were performed in triplicate. The primer sequences for the target genes shown in Supplementary Table 3 were purchased from Bioneer Company (Daejeon, Republic of Korea).

### Immunocytochemistry

The cells were fixed for 20 min with 4% paraformaldehyde and 0.1% Triton X-100 in PBS buffer. After washing with PBS, the fixed cells were incubated in a blocking solution (PBS containing 0.5% FBS) for 1 h at room temperature to reduce nonspecific antibody binding. The cells were treated with diluted primary antibodies in a blocking solution for 1 h at room temperature or overnight at 4 °C. After washing three times with PBS, the cells were incubated with Alexa Fluor-conjugated secondary antibodies (1000:1). The nuclei were stained with 4′,6-diamidino-2-phenylindole dihydrochloride (DAPI; Vector Laboratories; Burlingame, CA, USA; H-1200). The cells were imaged by using a Nikon Eclipse TE2000 fluorescence microscope. As indicated in the text, the neuronal cells were also imaged using confocal microscopy (LSM 510 Meta and 800; Zeiss, Göttingen, Germany) with a × 2 × , × 40, and × 100 objective, and the images were printed. The antibodies used for immunocytochemistry are listed in Supplementary Table 4.

### ssDNA Detection and Image Acquisition

To detect single-stranded DNA (ssDNA), the cells were fixed on ice with 4% PFA for 20 min and then with 80% methanol in PBS at − 20 °C overnight. The next day, the cells were washed and treated for 4 h at 37 °C with 200 µg/mL RNase (QIAGEN) and S1 nuclease (Promega) as indicated. Then, the cells were washed and blocked with a blocking solution (PBS containing 0.5% FBS), and then incubated overnight at 4 °C with the anti-ssDNA primary antibody. The following day, the cells were incubated with the secondary antibody for 2 h and then DAPI before mounting. Images of ssDNA were blindly captured by using confocal microscopy (LSM 510 Meta and 800; Zeiss, Göttingen, Germany) with a × 100 objective.

### Western Blot Analysis

For western blotting, the cells were harvested and lysed with RIPA buffer (150 mM NaCl, 20 mM Tris–HCl pH 7.4, 2 mM NaF, 2 mM EDTA, 5 mM sodium orthovanadate, 1% Triton X-100, 1 mM PMSF, protease inhibitor cocktail), incubated on ice for 10 min and then centrifuged at 13,000 rpm for 15 min, and the supernatant was collected. The protein concentration was determined using the BCA assay (Thermo Scientific, MA, USA). After quantification, the lysates were mixed with 2X sample buffer with β-mercaptoethanol and boiled at 95 °C for 10 min. The samples were subjected to SDS-PAGE and transferred to nitrocellulose membranes. The membranes were blocked with 5% nonfat skim milk in TBS with 0.1% Tween-20 (TBST) for 1 h and then incubated with primary antibodies (1:1000) at 4 °C overnight. Then, the membranes were washed 3 times with TBST for 10 min and incubated with secondary antibodies in TBST (1:3000) for 1 h at room temperature. After washing, ECL solution was added to the membrane, and the chemical luminescence was detected using a LAS-4000 (GE, WI, USA). The antibodies used for western blot analysis are listed in Supplementary Table 5.

### Immunoprecipitation

Cells were harvested and lysed with Pierce immunoprecipitation (IP) lysis buffer (Thermo scientific, MA, USA; 87,787) containing Halt Protease and Phosphatase Inhibitor Cocktail (Thermo scientific, MA, USA; 78,444). After quantification, lysates were used for immunoprecipitation using indicative antibodies and protein G-agarose beads (Roche, IN, USA; 11,719,416,001). After incubation with indicated antibodies and protein G-agarose beads over night at 4 °C, immunoprecipitates were washed three times with ice-cold 1 × Tris-buffered saline (TBS). The proteins were eluted with Laemmli sample buffer (Bio-Rad; #161–0747) and boiled at 95 °C for 10 min. Then, samples were subjected to western blotting using indicated antibodies.

Glutathione S-Transferase Pull Down.

293 T cells were cotransfected with expression vectors for p-glutathione S-transferase (GST)-tagged-BiP/GRP78 and FLAG tagged-TREX1 plasmids. GST was precipitated from cell lysates. The precipitates were western blotted with indicated antibodies.

### Duolink In Situ Proximity Ligation Assay

A proximity ligation assay (PLA) experiment was performed using a Duolink® in situ red kit according to the manufacturer’s instructions (Sigma-Aldrich). Briefly, the cells were fixed for 10 min with 4% paraformaldehyde and 0.1 Triton X-100 in PBS buffer. After washing with PBS, the fixed cells were blocked using Duolink® blocking solution for 30 min at 37 °C. Primary antibodies against GFP and BiP/GRP78 were diluted with Duolink® antibody diluent, applied to each cell line and incubated overnight at 4 °C. The cells were washed three times for 5 min each time with in Duolink® wash buffer A and incubated with Duolink® anti-mouse MINUS and anti-rabbit PLUS for 1 h at 37 °C. After washing three times, ligation and amplification reactions were performed for 30 min and 100 min at 37 °C, respectively. The cells were washed twice with Duolink® wash buffer B and then mounted with Duolink® mounting medium. The cells were examined using confocal microscopy (LSM 510 Meta and 800; Zeiss, Göttingen, Germany).

### Cell Death Assay

TUNEL assays were performed using the DeadEnd™ Colorimetric TUNEL System Kit (Promega; Catalogue, G7130) to confirm apoptosis in neuronal cells. Briefly, both control and TREX1-silenced neurons were fixed with 4% formaldehyde and then were incubated at room temperature for 30 min. Then, the cells were washed twice with PBS, followed by permeabilization in 0.2% Triton X-100 solution on ice for 5 min. Next, the steps of apoptosis detection were followed according to the manufacturer’s protocol. The cells were mounted in permanent mounting medium and imaged by using a Nikon Eclipse TE2000 fluorescence microscope. The data are expressed as the ratio of TUNEL-stained cells to the total number of neurons.

To measure the apoptosis of cultured primary neurons, propidium iodide (20 µg/ml) was added to the neuronal cultures and incubated for 4–5 min, followed by fixation with 4% paraformaldehyde solution and visualization using fluorescence microscopy.

### Detection and Visualization of Intracellular Ca^2+^

For the fluorescence detection of intracellular Ca^2+^, the cells were grown in the wells of 96-well plates. The cells were incubated with the fluorescent calcium indicator Fluo-4-AM (5 µM; Invitrogen) in HBSS at 37 °C for 45 min. The labeled cells were washed twice with HBSS and used for intracellular Ca^2+^ detection. The fluorescence measurement of the intracellular Ca^2+^ levels was performed with the use of a Spark Multimode Microplate Reader (Tecan, Switzerland). The Fluo-4-Ca^2+^ complex (526 nm) was recorded for the indicated times. All the measurements were normalized by the fluorescence of the unlabelled cells. In the experiments, where indicated, 2 μM thapsigargin, an inducer of Ca^2+^ release from the ER, was added to assess the ER Ca^2+^ stores, and the data were recorded for ~ 180 s. The data were quantified as either the increment in *f*_max_/*f*_0_ for the first peak or the area under the curve (AUC) for all the peaks.

For the visualization of intracellular Ca^2+^, the cells were grown on coverslips in.

12-well plates and then treated with 5 µM Fluo-4-AM (Invitrogen) for 45 min in complete media at 37 °C. To track the ER Ca^2+^ levels, the cells were costained with the fluorescent ER-Tracker Blue-White DPX dye (3 µM; Life Technologies) and Fluo-4-AM for 45 min at 37 °C. The cells were washed twice with HBSS and then mounted on the stage of an inverted confocal microscope (LSM 510 Meta and 800; Zeiss, Göttingen, Germany) equipped with × 40 and × 100 objective.

### Clinical Specimens of Korean HSP Patients

The study protocol was approved by the Institutional Review Board of Samsung Medical Center, Seoul, Korea, between 2014 and 2016. A total of 109 samples (83 affected individuals and 26 healthy controls) were collected from 60 families with HSP, the members of which displayed features consistent with either pure (*n* = 71) or complicated (*n* = 12) spastic paraplegia. All the patients underwent neurologic and genetic evaluations after giving informed consent. All the individuals were seen by a board-certified neurologist. The individuals who displayed clinical features attributable to disorders other than HSP were excluded from the study.

### Exome Sequencing Data Analysis

Whole-exome sequencing (WES) was performed using the HiSeq 2000/2500 platform within the Core Facility Management Center of the Korea Research Institute of Bioscience and Biotechnology (KRIBB). The sequencing libraries were prepared from primary DNA extracted from the leukocytes of blood samples using the TruSeq library preparation kit (Illumina, San Diego, CA, USA) following the manufacturer’s protocol. The Nextera Rapid Capture Exome kit (Illumina) was used to selectively amplify the coding regions of the genome according to the manufacturer’s protocol. The captured libraries were sequenced using a HiSeq 2000/2500 sequencer (Illumina), and 2 × 100 bp were utilized for paired-end sequencing according to the manufacturer’s recommendations. Image analysis and base calling were performed using the Illumina pipeline. The sequencing data were mapped to the human reference genome (GRCh37, UCSC hg19) using the Burrows-Wheeler Aligner software. PCR duplicates were removed using Picard software. The single-nucleotide variants (SNVs) and insertions-deletions (INDELs) were identified based on the filtered variants with a mapping quality score ≥ 20 using the Genome Analysis Toolkit (GATK, version 3.6) software from the Broad Institute. The ANNOVAR software was used to functionally annotate variants.

### Extrachromosomal DNA Extraction and Deep Sequencing

The extrachromosomal DNA was extracted from both control and TREX1-silenced neuronal cells using the modified Hirt protocol [43]. To extract the extranuclear DNA, approximately 1 × 10^7^ cells were treated with a trypsin–EDTA solution, pelleted, and washed with DPBS. The cells were resuspended in 250 µL Buffer A (50 mM Tris–HCl; pH 7.5, 10 mM EDTA, supplemented with 100 µg/mL RNase A). The cells were then lysed by the addition of 250 µL Buffer B (1.2% sodium-dodecyl sulfate; SDS). The suspension was gently mixed by inversion and incubated at room temperature for 5 min to ensure complete lysis. The cellular debris and chromosomal DNA were precipitated by the addition of 350 µL Buffer C (3 M CsCl, 1 M potassium acetate, and 0.67). The solution was chilled on ice for 15 min. After centrifugation for 15 min at 14,000 × *g*, the supernatant containing extrachromosomal DNA was collected and column purified (QIAprep^R^ Spin Column; Catalogue 27,104; QIAGEN; Germany). Single-cycle PCR was performed to convert the ssDNA into dsDNA to be sequenced. The samples were sequenced at the Macrogen, Seoul, Republic of Korea.

### Characterization of Extrachromosomal DNA

The extrachromosomal DNA was extracted from both control and TREX1-silenced neuronal cells using the abovementioned protocol, and the sequencing libraries were prepared by the TruSeq Nano DNA Kit (Illumina, San Diego, CA, USA). Deep sequencing was performed on an Illumina platform. The high-quality sequencing reads of each sample were mapped to the human reference genome (build Hg38) using the BWA-MEM software, and SAM mapping files were obtained [44]. Next, we used SortSam in GATK4 to convert the SAM file into sorted mapping BAM files. For the correlation of the mapped reads with the annotated molecules in Hg38, we created a nonredundant GTF annotation file of repetitive elements for RepeatMasker [45]. GTF annotation and sorted BAM files were compared using the HTSeq-count software in the HTSeq package to count the sequencing reads of each repetitive element [45]. Finally, the counted reads per sample were RPKM (reads per kilobase per million) normalized by DESeq, a Bioconductor software package (https://bioconductor.org/ packages/release/

bioc/html/DESeq.html).

### Statistical Analysis

All experiments were performed in triplicate. The error bars represent the SEM. Unpaired, two-tailed Student’s *t* test was used for comparisons between two groups. One-way ANOVA was performed for multigroup comparisons. Differences with *p* values of less than 0.05 were considered significant. The significance levels are expressed as follows: *p < 0.05, **p < 0.01, and ***p < 0.001.

## Supplementary Information

Below is the link to the electronic supplementary material.

Supplementary file1 (DOCX 3795 KB)

## Data Availability

All data generated or analyzed during this study are included in this article and its supplementary information files.
