# Supplementary material for: TREX1 Deficiency Induces ER Stress-Mediated Neuronal Cell Death by Disrupting Ca2+ Homeostasis
Source: Mol Neurobiol. 2022 Jan 7;59(3):1398–418. doi: 10.1007/s12035-021-02631-3 (PMC8882114; doi:10.1007/s12035-021-02631-3)
Supplement: Supplementary file 1 — Supplementary file1 (DOCX 3795 KB) [file 12035_2021_2631_MOESM1_ESM.docx]

**TREX1 deficiency induces ER stress-mediated neuronal cell death by disrupting Ca^2+^ homeostasis**

Debasish Halder^1^, Su-Jin Jeon^1,3^, Ji-Yong Yoon^1,2^, Jeong-Ju Lee^1,2^, Soo Young Jun^1,2^, Min-Hyuk Choi^1,3^, Bohyeon Jeong^1^, Duk-Hyun Sung^4^, DaYong Lee^1^, Byoung-Joon Kim^5^, Nam-Soon Kim^1,2,3,*^

*^1^Rare Disease Research Center, ^2^Genome Research Center, Korea Research Institute of Bioscience and Biotechnology, 34141 Daejeon, Republic of Korea; ^3^Department of Functional Genomics, Korea University of Science and Technology, 34113 Daejeon, Republic of Korea; ^4^Department of Physical and Rehabilitation Medicine, ^5^Department of Neurology Sungkyunkwan, Sungkyunkwan University School of Medicine, Samsung Medical Center Gangnam-gu, 06351 Seoul, Republic of Korea*

**^*^Correspondence:** Nam-Soon Kim

Rare Disease Research Center, Korea Research Institute of Bioscience and Biotechnology (KRIBB), Daejeon 34141, Republic of Korea

Tel.: +82-42-879-8112, Fax: +82-42-879-8119

E-mail: [nskim37@kribb.re.kr](mailto:nskim37@kribb.re.kr)

**Running tile: TREX1 deficiency induces ER stress-mediated cell death**

**Supporting Information:**

**Supplementary Fig 1.**

**Supplementary Fig 2.**

**Supplementary Fig 3.**

**Supplementary Fig 4.**

**Supplementary Fig 5.**

**Supplementary Table 1.**

**Supplementary Table 2.**

**Supplementary Table 3.**

**Supplementary Table 4.**

**Supplementary Table 5.**


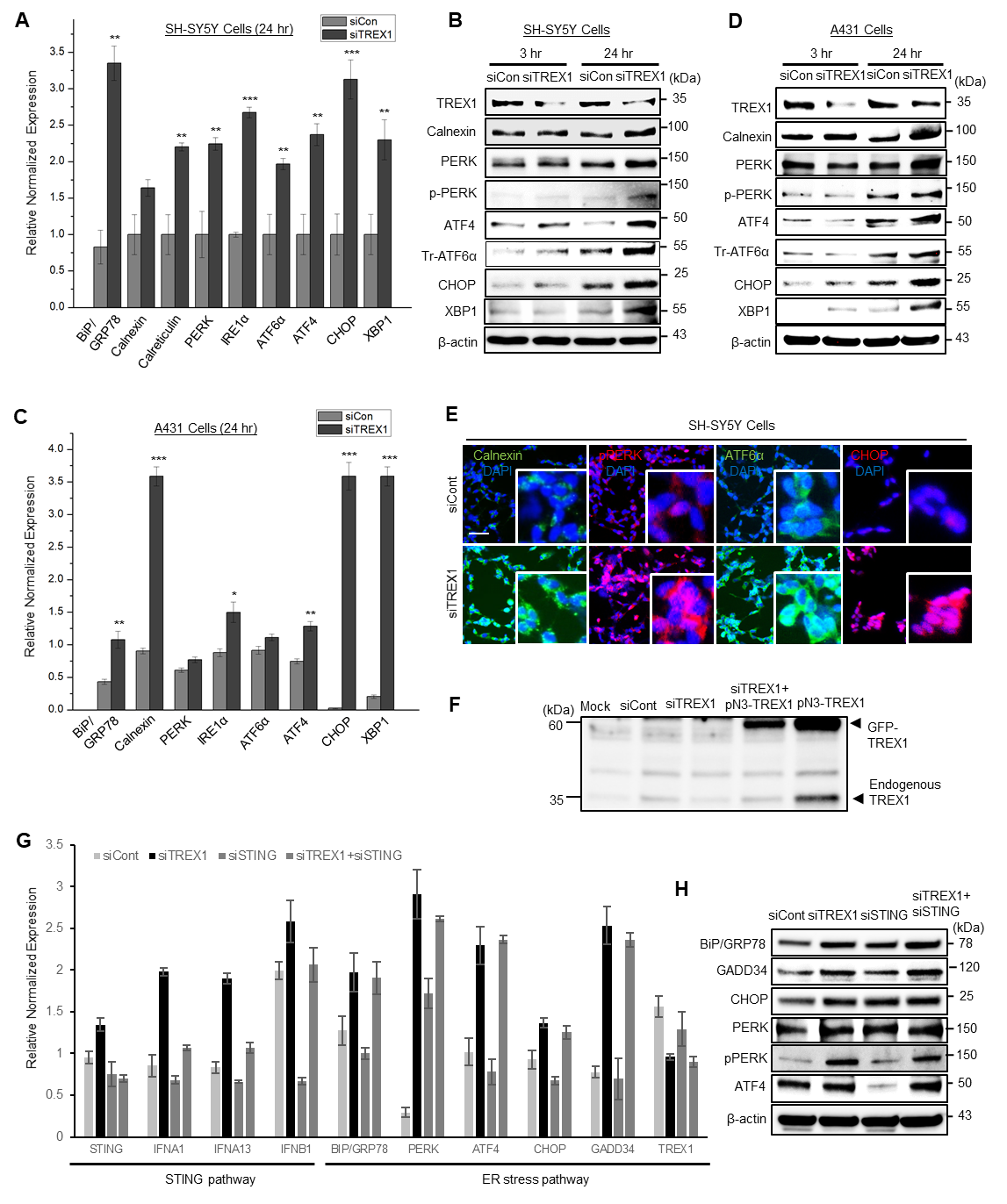


**Supplementary Fig. 1. TREX1 knockdown activates the ER stress and UPR pathways.** SH-SY5Y and A431 cells were transfected with siCont and siTREX1. The cells were harvested at the indicated times (3 hr and 24 hr). (A) Quantitative RT-PCR analysis of UPR pathway genes in TREX1-knockdown SH-SY5Y cells. (B) Western blot analysis of UPR proteins in TREX1-knockdown SH-SY5Y cells. (C) The mRNA levels of UPR pathway genes in TREX1-knockdown A431 cells. (D) Western blot analysis of UPR proteins in A431 cells. (E) Immunocytochemical analysis of UPR proteins in TREX1-knockdown SH-SY5Y cells. The cells were stained with the indicated antibodies and DAPI (blue, nuclei). Scale bar: 50 µm. (F) Western blot detection of the TREX1 protein in the indicated samples. (G, H) SH-SY5Y cells transfected with either siTREX1 or siSTING or a combination of both underwent neuronal differentiation. The expression levels of the STING-IFN pathway and ER stress signalling genes were measured by quantitative RT-PCR analysis (G) and western blotting (H). The error bars show the SEM. *, P < 0.05; **, P < 0.01; ***, P < 0.001; Student’s *t*-test. The data shown in all the panels are representative of three independent experiments.


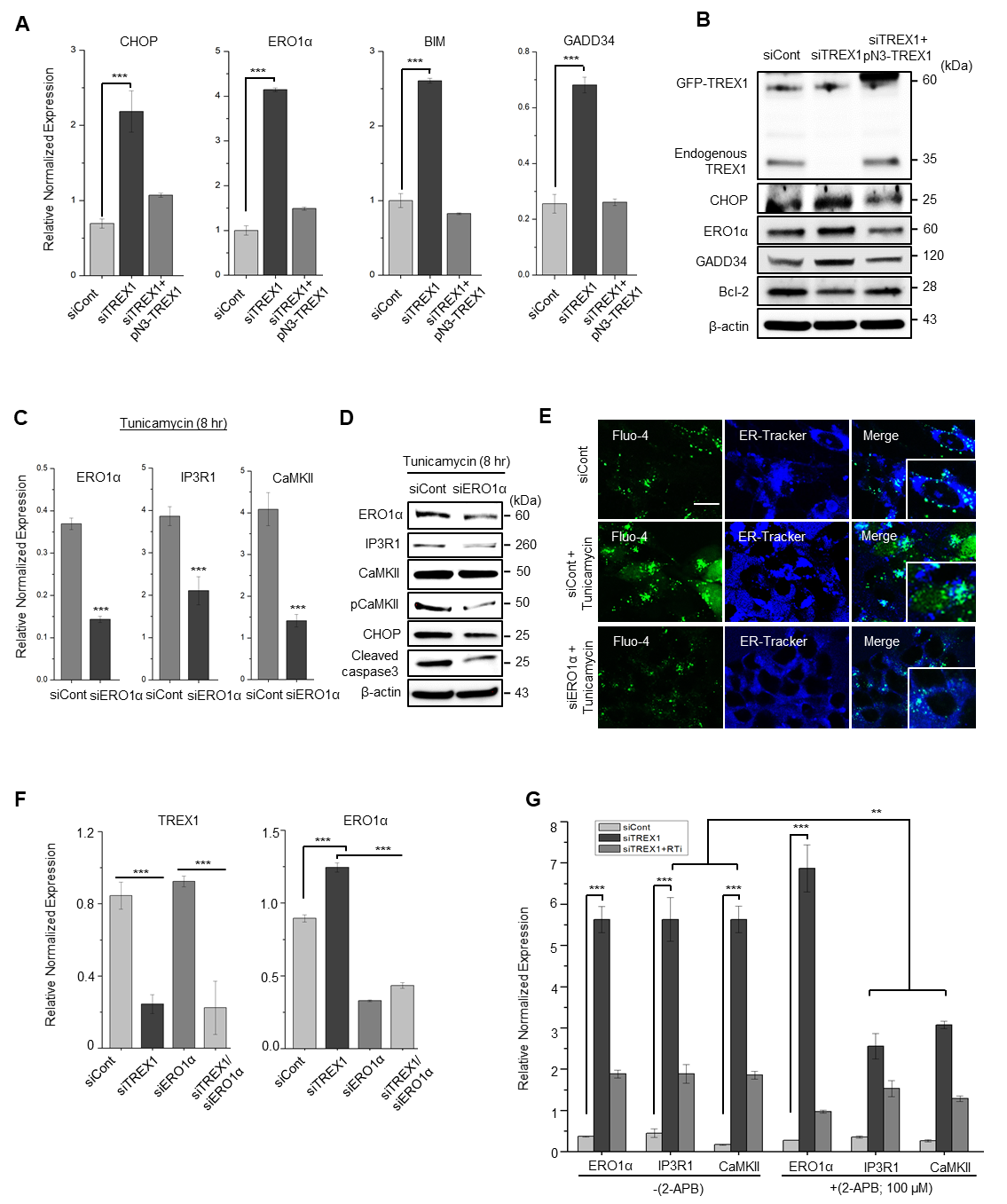


**Supplementary Fig. 2. TREX1 knockdown activates CHOP targets that are involved in ER Ca^2+^ homeostasis.** (A, B) SH-SY5Y cells transfected with siTREX1 or siCont were transfected with/without pN3-TREX1 and underwent neuronal differentiation. The transcriptional levels of CHOP and its targets were measured by quantitative RT-PCR analysis (A) and Western blotting (B). (C-E) SH-SY5Y cells were transfected with either control or ERO1α siRNA and incubated for 8 hr in the absence or presence of the ER stress inducer tunicamycin (5 μg/ml). The expression level of the ER Ca^2+^-releasing channel protein IP3R1 and its downstream partner CaMKII in ERO1α-knockdown cells was examined by quantitative RT-PCR (C) and Western blot analysis (D). Both the IP3R1 and CaMKII levels were reduced in the ERO1α-knockdown cells. (E) The intracellular Ca^2+^ levels in tunicamycin-treated ERO1α-knockdown cells were visualized with Fluo-4 (5 µM) by confocal microscopy. (F) The expression levels of the TREX1 and ERO1α genes in the indicative samples. (G) The mRNA levels of ER Ca^2+^ regulatory pathway genes were examined in TREX1-knockdown cells treated with an IP3R channel blocker (2-APB, 2 μM) by quantitative RT-PCR analysis. The error bars show the SEM. **, P < 0.01; ***, P < 0.001; Student’s *t*-test for two groups and one-way ANOVA was performed for multigroup comparisons. The data shown in all the panels are representative of three independent experiments.


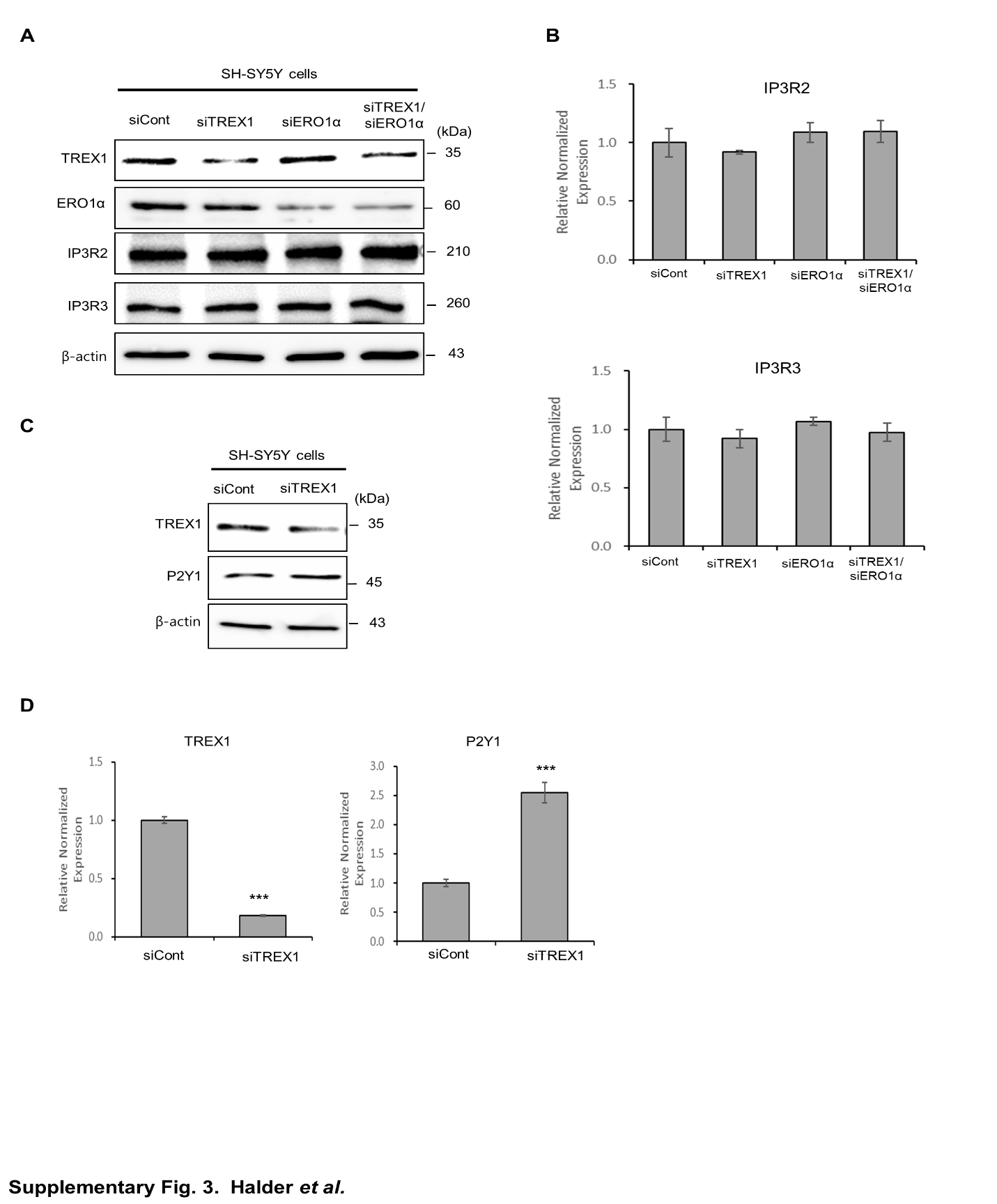


**Supplementary Fig. 3 The effects of TREX1 knock down on IP3R2/3 and P2Y1.** SH-SY5Y cells transfected with siTREX1 or siCont were allowed to neuronal differentiation. The protein and transcriptional levels of IP3R2 and IP3R3 were measured by Western blotting (A) and quantitative RT-PCR analysis (B). The expression level of TREX1 and P2Y1 was determined by western blot analysis (C) and real time PCR (D).


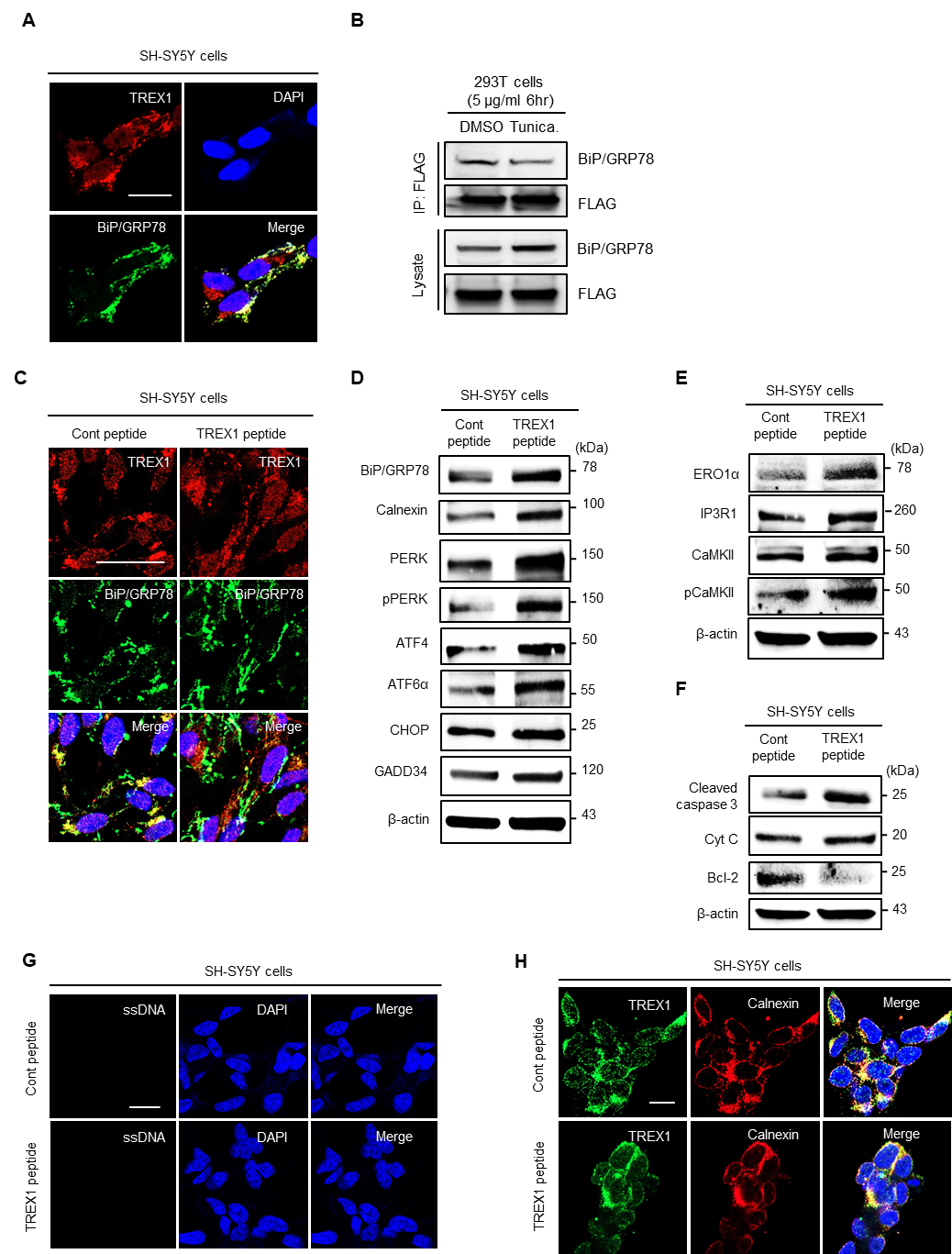


**Supplementary Fig. 4. A synthetic peptide blocking the interaction between TREX1 and BiP/GRP78 induces ER stress signalling.** (A) Endogenous expression of TREX1 and BiP/GRP78 in SH-SY5Y cells. (B) 293T cells were treated with tunicamycin (5 μg/ml, 6 hr), and cell lysates subjected to immunoprecipitation with anti-FLAG antibodies, and the proteins were measured by Western blot analysis. (C-H) SH-SY5Y cells treated with control and TREX1 mimic peptides underwent neuronal differentiation. Immunocytochemical analysis showing decreased colocalization of TREX1 and BiP/GRP78 in SH-SY5Y cells treated with a TREX1 mimic peptide (C). The expression levels of UPR pathway markers (D), Ca^2+^ pathway markers (E), and cell death markers (F) in the peptide-treated cells were examined by Western blot analysis. (G) The accumulation of ssDNA in TREX1 peptide-treated cells was determined by immunostaining with an antibody against ssDNA. (H) The localization of TREX1 in peptide-treated cells was determined by immunostaining with antibodies against TREX1 (green) and Calnexin (an ER marker; red). Cont: control peptide. TREX1 peptide: TREX1-mimicking peptide. The yellow colour indicates colocalization of BiP/GRP78 and PERK. Scale bar: 20 µm.


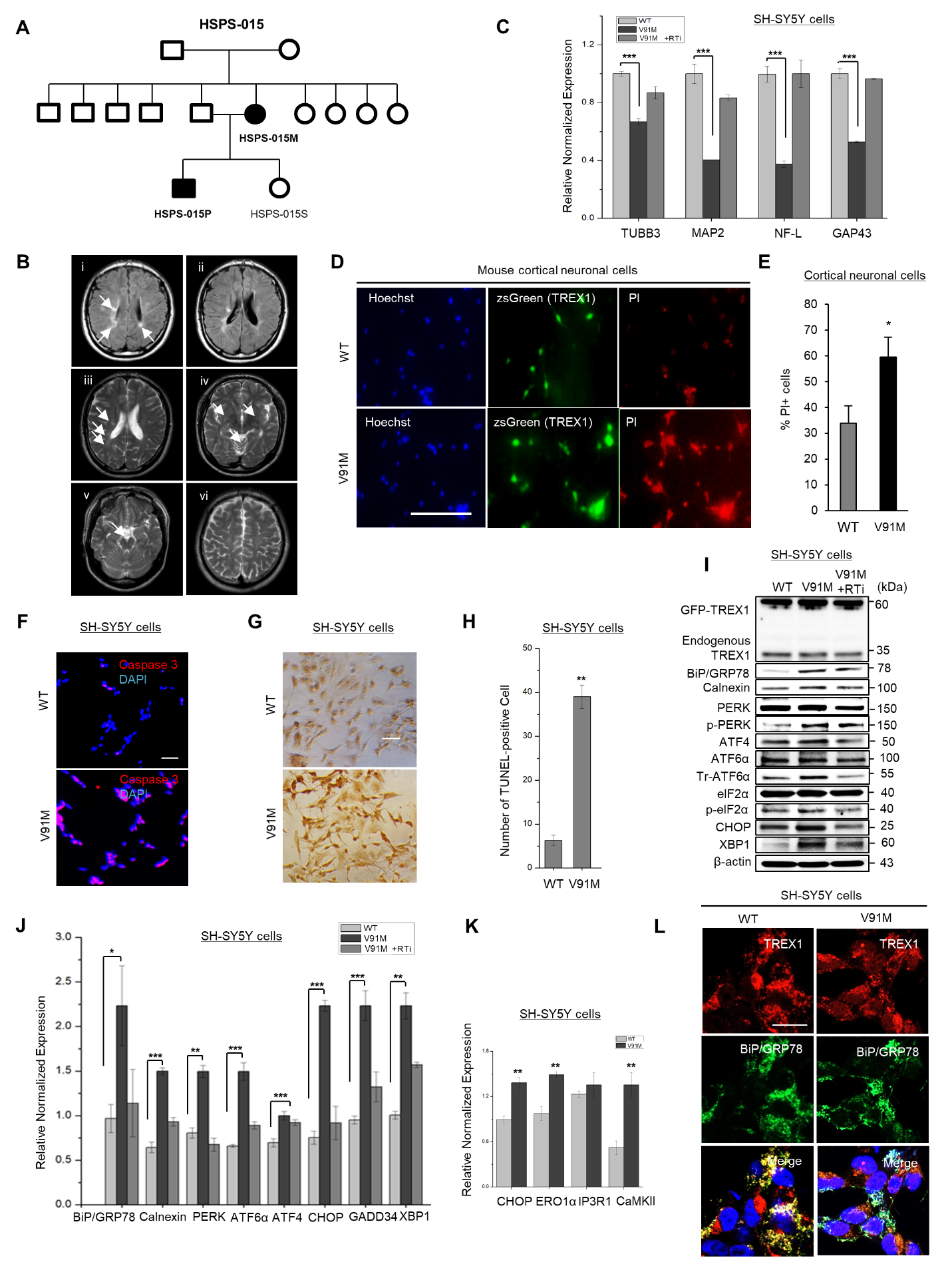


**Supplementary Fig. 5. TREX1 mutation identified in a Korean family with HSP impairs neuronal development by activating ER stress and UPR signalling.** (A) The pedigree of a family with an autosomal-dominant missense mutation (cG271A, pV91M) in the TREX1 gene. Three members of the family, which are marked in red, were recruited for whole-exome sequencing analysis. M, mother (born in 1965); P, proband (born in 1992); and S, sibling (born in 1994). (B) Brain MRI scans of a patient diagnosed with HSP. Images showing white matter abnormalities of the periventricular (i, iii) and internal capsule (ii, iv) in the brain of the patient. (v) T2 weighted image showing atrophic midbrain. (vi) T axial polymicrogyria. (C-K) SH-SY5Y cells or mouse primary cortical neurons were transfected with plasmids expressing mutant (V91M) and wild-type TREX1 and allowed to undergo differentiation. The transcriptional levels of neuron-specific markers in differentiated SH-SY5Y cells were examined by quantitative RT-PCR analysis (C). The surviving primary cortical neurons were scored based on propidium iodide (PI) staining of the TREX1-mutant neurons. Representative images (D) and graphical presentation (E) of the PI-positive cells. The cells were counterstained with Hoechst 33258. Scale bar: 100 μm. The apoptosis process in differentiated SH-SY5Y cells was assessed by immunostaining with an anti- cleaved caspase 3 antibody (F) and TUNEL assay (G, H). (I) The expression levels of UPR pathway genes in differentiated SH-SY5Y cells were examined by Western blotting (I) and quantitative RT-PCR analysis (J). (K) The mRNA levels of CHOP and its downstream ERO1α-IP3R-CaMKII pathway genes in differentiated SH-SY5Y cells were measured by quantitative RT-PCR analysis. (L) Immunocytochemical analysis showing decreased colocalization of TREX1 and BiP/GRP78 in TREX1-deficient cells. Scale bar: 20 µm. The error bars show the SEM. *, P < 0.05; **, P < 0.01; ***, P < 0.001; Student’s *t*-test for two groups and one-way ANOVA was performed for multigroup comparisons. The data shown in all the panels are representative of three independent experiments.

**Supplementary Table 1. Clinical Characteristics of Patients**

| **Characteristics** | **Patient ID** | |
| --- | --- | --- |
|  | **HSPS-015P** | **HSPS-015M** |
| Sex | Male | Female |
| Age | 22 | 49 |
| Onset age | 20 | 23 |
| Mutation | c.271G > A | c.271G > A |
| SPRS | 3 | 36 |
| Phenotype | Pure | Pure |
| LE spasticity | Y | Y |
| LE weakness | Y | Y |
| MRI | Brain atrophy, T2 white matter hyperintensities | T2 white matter hyperintensities |
| SPAST | negative | negative |
| ATL1 | negative | negative |

**Abbreviation:** SPRS, Spastic Paraplegia Rating Scale; LE, Lower extremity

**Supplementary Table 2. Reagents and Chemicals**

| **Reagent and Resource** | **Source** | **Identifier** |
| --- | --- | --- |
| Lamivudine (3TC) | Sigma-Aldrich | L1295 |
| Stavudine (d4T) | Tocris Bioscience | 1A/214432 |
| Thapsigargin (ThG) | Cayman Chemical | 10522 |
| Retinoic acid (RA) | Sigma-Aldrich | R2625 |
| Brain-derived neurotrophic factor (BDNF) | Sigma-Aldrich | SRP3014 |
| Lipofectamine RNAiMAX reagent | Invitrogen | 13778150 |
| Lipofectamine 2000 | Invitrogen | 1668019 |
| FUGENE 6 Transfection reagent | Promega | E2691 |
| S1 nuclease | Promega | E576B |
| RNAeasy Mini Kit | QIAGEN | 74136 |
| iScriptTM cDNA synthesis kit | Bio-rad laboratories Inc. | 1708891 |
| iQ SYBR Green Supermix | Bio-rad laboratories Inc. | 1708882 |
| DeadEndTM Colorimetric TUNEL System Kit | Promega | G7130 |
| Fluo-4-AM | Biotium | 50018 |
| ER-Tracker Blue-White DPX dye | Life Technologies | E12353 |
| QIAprepR Spin Cloumn | QIAGEN | 27104 |
| TruSeq Nano DNA Kit  Subcellular Protein Fractionation Kit for Cultured Cells | Illumina  Thermo Scientific™ | 20015965  78840 |

**Supplementary Table 3. Primers used for qRT-PCR analysis**

| **Gene Name** | **Forward primer** | **Reverse primer** |
| --- | --- | --- |
| **Human** |  |  |
| TREX1 | GGCTCCCAGCAGTGTGTAAG | GCTCAGACCTGTGATCTCGC |
| TUBB3 (Tuj1) | CTGGCCATCCAGAGCAAGAA | CGTACATCTCGCCCTCTTCC |
| MAP2 | TAGACCTAAGCCATGTGACA | AGAACCAACTTTAGCTTGGG |
| Neurofilament-L | CTGGAAATCGAAGCATGCCG | GCGGGTGGACATCAGATAGG |
| BiP/GRP78 | CCCGAAAAGCATTTGGGCAG | AACCTAGGTAGGTGTGATTTGTGT |
| Calreticulin | GACTTCCTGCCACCCAAGAA | CTCAGCGTATGCCTCATCGT |
| IP3R1 | AAGCGAGTTCCTGTTCTCCG | ACATCTCCTACTCCACCCCC |
| ERO1α | TCTTCGAGCGCCCAGATTTT | GAAGCTTTCCCCACAGACGA |
| Bcl-2 | TCATGTGTGTGGAGAGCGTC | GGGCCAAACTGAGCAGAGTC |
| CHOP | GAATGAACGGCTCAAGCAGG | GGCAGACAAGAACGAGGTTG |
| Calnexin | AGCCCTTCCTGTTTGACACC | CTGGCTTGACAGCTTCTGGA |
| PERK | AGTGACTGCAATGGACCAGG | CTTGGCAAAGGGCTATGGGA |
| XBP1 | TCCCCCTTTTTGGCATCCTG | GGTGGAAAAGCCTTCAGTCAC |
| ATF6α | CAGCAGGAACTCAGGGAGTG | TCAGGGATGGTGCTGACAAC |
| CHOP | GAATGAACGGCTCAAGCAGG | GGCAGACAAGAACGAGGTTG |
| IRE1α | TGGGTGGCCTTCATCATCAC | AACATGCCCCGGTACACAAT |
| CaMKII | CTCAAGGGAGCCATCCTCAC | GGGCTTGACTCCATCTGCTT |
| GADD34 | GCTGAGTCAGACCCACATCC | TCCGGATCATGAGTAGGGGT |
| ATF4 | CCGTGAGCGTCCATTTTGTG | GTAGGAGGCCCCTAACCCTA |
| BIM | GCTACCAGATCCCCGCTTTT | CAATACGCCGCAACTCTTGG |
| TREX1 | CTGGATGGTGCCTTCTGTGT | AGATCCTTGGTACCCCTGCT |
| GAP43 | GTGCCACTAAAGCTTCCACT | GGGCACTTTCCTTAGGTTTG |
| GAPDH | GACCACAGTCCATGCCATCA | GTCAAAGGTGGAGGAGTGGG |
| **Mouse** |  |  |
| Trex1 | CTTCCTCAGCCACACTGCTG | CCTGGAGCAATGCACAGAGA |
| ERO1α | TGGCTATGTCCTCTGTCCCTC | TGTAAAGCCTTGAACGGTGC |
| CaMKII | CCCCAAAGTCACAGAGCCAT | ACGTGTCGTCTTCCACTGTC |
| IP3R1 | TAGGAGCTGTCCCCTTAGGA | GCTTCGGAACTCTGCATACG |
| Calnexin | GCTAGGGAGAATGAATTGCCG | ATGGAGCAGTCTCTAGGGCA |
| BiP/GRP78 | TGTGTGTGAGACCAGAACCG | TAGGTGGTCCCCAAGTCGAT |
| PERK | CCGAATCACCTGACGGTTCA | GAGGGTTGCTTGTTTGCAGG |
| Chop | GCAGCGACAGAGCCAGAATA | TTCCTCCTCTTCCTCCTGGG |
| eIF2α | CACGGTGCTTCCCAGAGAAT | GGCAAACTGCTTTTGGGAGG |
| ATF4 | CCTATAAAGGCTTGCGGCCA | ATCTCGGTCATGTTGTGGGG |
| ATF6α | GGTGGAAGTGGGAAGATCGG | GCCACAGGTCCTCTTTAGGC |
| Gapdh | CCCTTAAGAGGGATGCTGCC | ACTGTGCCGTTGAATTTGCC |

**Supplementary Table 4. Antibodies used for immunocytochemistry**

| **Antibody name** | **Manufacturer** | **Cat. No.** |
| --- | --- | --- |
| TREX1 | Abcam | ab185228 |
| TUBB3 (Tuj1) | Abcam | ab18207 |
| MAP2 | Abcam | ab11267 |
| Neurofilament-L (C28E10) | Cell Signaling | 2837P |
| Caspase 3 | Cell Signaling | 9665 |
| XBP-1S (D2C1F) | Cell Signaling | 12782S |
| α-Tubulin | Santa Cruz | sc-8035 |
| Ac-α-Tubulin (D20G3) | Cell Signaling | 5335S |
| GM-130 | Abcam | ab169276 |
| ssDNA (F7-26) | Enzo | aLX-804-192 |
| Calnexin  Calnexin | Abcam  Abcam | ab31290  ab219644 |
| PERK | Cell Signaling | 3192S |
| ATF6α | Santa Cruz | sc-48381 |
| CHOP | Cell Signaling | 5554S |
| Goat anti-Mouse lgG-Alexa Fluor 488 | Life technologies | A11001 |
| Goat anti-Mouse lgG-Alexa Fluor 568 | Life technologies | A11004 |
| Goat anti-Rabbit lgG-Alexa Fluor 488 | Life technologies | A11008 |
| Goat anti-Rabbit lgG-Alexa Fluor 568 | Life technologies | A11011 |
| Goat anti-Goat lgG-Alexa Fluor 568 | Life technologies | A11086 |

**Supplementary Table 5. Antibodies used for western blot analysis**

| **Antibody name** | **Manufacturer** | **Cat. No.** |
| --- | --- | --- |
| TREX1 | Abcam | ab185228 |
| TUBB3 (Tuj1) | Abcam | ab18207 |
| MAP2 | Abcam | ab11267 |
| Neurofilament-L (C28E10) | Cell Signaling | 2837P |
| BiP/GRP78  BiP/GRP78 | Cell Signaling  Santa Cruz | 3183S  Sc-166490 |
| Calreticulin | Abcam | Ab22683 |
| Calnexin | Abcam | ab31290 |
| ATF6α | Santa Cruz | sc-48381 |
| CHOP | Cell Signaling | 5554S |
| Calnexin | Abcam | Ab10286 |
| PERK | Cell Signaling | 3192S |
| p-PERK (T981) | Santa Cruz | sc-32577 |
| p-eIF2α (S51) | Cell Signaling | 9721 |
| eIF2α | Cell Signaling | 9722 |
| PARP | Cell Signaling | 9542 |
| ATF4 | Santa Cruz | sc-390063 |
| XBP1 | Cell Signalling | 83418 |
| Cytochrome C | Abcam | Ab76107 |
| Caspase 9 | Cell Signaling | 9502S |
| GADD34 | Santa Cruz | sc-8327 |
| DR5 | Santa Cruz | sc-166624 |
| Caspase 3 | Cell Signaling | 9665 |
| Cleaved caspase 3 (ASP175) | Cell Signaling | 9664 |
| Bcl-2 | Cell Signaling | 15071S |
| Bcl-xL | Cell Signaling | 2762S |
| CaMKII | Santa Cruz | sc-5306 |
| p-CaMKII | Santa Cruz | sc-32289 |
| ERO1α (YW-8) | Santa Cruz | sc-100805 |
| IP3R1 (E-8) | Santa Cruz | sc-271197 |
| α-Tubulin | Santa Cruz | sc-8035 |
| Ac-α-Tubulin (D20G3) | Cell Signaling | 5335S |
| β-actin | Santa Cruz | sc-47778 |
| Laminin B1 | Santa Cruz | Sc-374015 |
| GST | Santa Cruz | Sc-138 |
| Goat-anti mouse IgG-HRP | Santa Cruz | sc-2031 |
| Goat- anti rabbit IgG-HRP  GFP  Anti-FLAG  Normal Mouse IgG  Normal Rabbit IgG | Santa Cruz  Santa Crutz  Sigma Aldrich  Santa Cruz  Santa Cruz | sc-2004  sc-8334  F1804  sc-2025  sc-2027 |
